# An illustrated guide to seeds found in nests of the Florida harvester ant, *Pogonomyrmex badius*

**DOI:** 10.1371/journal.pone.0171419

**Published:** 2017-03-01

**Authors:** Walter R. Tschinkel, Daniel J. Domínguez

**Affiliations:** Department of Biological Science, Florida State University, Tallahassee, Florida, United States of America; Arizona State University, UNITED STATES

## Abstract

The Florida harvester ant, *Pogonomyrmex badius* collects the seeds of many plant species and stores them in underground nest chambers for later consumption. Seeds taken from multiple nests in 1989, 2014 and 2015 were separated by size and species and identified through published keys, comparison with herbarium specimens and with identified seed collections. Harvester ants stored at least 58 species of seeds from 20 plant families in their chambers. This paper presents images of each seed species in several aspects, their relative abundance in *P*. *badius* nests, their size relative to the smallest, and links to online data and images of the parent plant species, as well as to herbarium specimens. A number of seeds and plant families present at the site were not found in ant nests. These data and images will be valuable for future studies and experiments to untangle the choices the ants make in relation to what the plants and the seasons offer them.

## Introduction

The ability to collect, store and consume seeds has evolved independently more than 18 times among the genera of ants [1,2]. Seed harvesting ants are often associated with arid or semi-arid habitats. Most of the 29 North American species of *Pogonomyrmex* harvest and consume seeds [2], some to such an extent that it was claimed that they compete with seed-eating mammals [3], although later work cast doubt on this claim (reviewed in [2]). Usually, ants collect multiple seed species, ranging from a few to about 45[4–6]. For most harvesting ant species, seeds form only part of their diet, the remainder consisting of insects, fungus, plant material etc., but a few feed almost exclusively on seeds[1,7–10]. Seed preferences have been reported to be correlated to body size, abundance, nutritional content, toxicity and novelty [11–16].

Most harvesting ants store the seeds in underground chambers. For *P*. *badius*, these chambers are located between about 40 and 100 cm below ground, and may contain (in the aggregate) up to 300,000 seeds weighing half a kg [17,18]. Seed collection is usually seasonal [9,19, 20], and while it has been suggested that some species may overwinter without seeds [7], this claim is contradicted by Lavigne [5].

Although early observers sometimes noted germinating seeds in the nests of harvesting ants, the possibility that the ants might actually require some seeds to germinate in order to be able to eat them was never carefully investigated until the recent work of Tschinkel and Kwapich [21]. Through experiments and observations, Tschinkel and Kwapich [21] showed that *P*. *badius* workers cannot open seeds larger than 1 to 1.4 mm wide, that they readily use germinated seeds, feeding these to larvae, that seeds actually germinate within the subterranean storage chamber are rapidly removed by workers to be fed to larvae, and that seed germination rates are related to the seed species, the season and temperature.

This paper is a companion paper to that of Tschinkel and Kwapich [21] for the purpose of providing identifications and images of the 58 species of seeds taken from *P*. *badius* nests. In addition, we also provide identifications and images of some other seeds found at the study site, Ant Heaven, but not within *P*. *badius* nests.

## Materials and methods

### Study site

The study population of Florida harvester ant, *P*. *badius*, is located in a 23 ha site (latitude 30.3587, longitude -84.4177) about 16 km southwest of Tallahassee, Florida, USA, within the sandhills ecotype of the Apalachicola National Forest. The site, Ant Heaven, consists of excessively drained sandy soil occupying a slope to a wetland and stream, causing its water table to be depressed (>5 m at the maximum), thereby making it suitable for *P*. *badius* and *Solenopsis geminata*, as well as several drought-resistant species in the genera *Opuntia* and *Nolina*. The forest consists of longleaf pines (*Pinus palustris*) planted ca. 1975, turkey oak (*Quercus laevis*), bluejack oak (*Quercus incana*), occasional sand pines (*Pinus clausa*) and sand live oak (*Quercus geminata*). Because the soil had been disturbed in the early 1970s, the natural ground cover of wiregrass (*Aristada stricta*) is absent, replaced by broomsedge (*Andropogon* spp.) and several other successional species of grasses, herbs and shrubs. The same disturbance may have helped establish this dense population of *P*. *badius*, whose nests are easily spotted because the ants decorate the excavated soil disc with a layer of charcoal bits (mostly the ends of burned pine needles). The black charcoal contrasts sharply with the light-colored sand or litter.

This project was carried out under US Forest Service, Apalachicola National Forest permit number APA56302, Expiration Date: 12/31/2017. *Pogonomyrmex badius* is not a protected species.

### Seed collection and preparation

Colonies of *P*. *badius* were excavated by digging a pit next to the nest and exposing the horizontal chambers one by one by lifting off the soil with a large trowel, whereupon seeds, ants and other contents were collected [17, 18]. Seeds were mostly found in dedicated chambers between about 30 and 100 cm below ground. The seeds used in this study were collected from 31 nests excavated in 1989 by WRT, nine in 2014 and eleven in 2015. All seeds were stored dry in the laboratory.

Seeds were separated into size classes with U.S. Standard Testing sieves No. 8–35, and the proportion of the total weight in each size class was computed. The more common seed species of all size classes were separated for determination of their mean weights. Four size classes of the 2014 seeds were used in a series of experiments on the consumption of germinating and non-germinating seeds. The results of these studies can be found in Tschinkel and Kwapich [21].

### Seed identification and imaging

Several inputs helped identify seeds. Two illustrated manuals with keys for identifying seeds important as food for wild quail [22, 23] were primary sources. Initial identifications were checked by comparing with seeds taken from identified herbarium specimens in the R.K. Godfrey Herbarium at Florida State University. Some were also compared with seeds in the seed collection of the University of Florida Herbarium in Gainesville, Florida. In addition, seed-bearing plants were collected at the study site, identified in the Godfrey Herbarium, and their seeds compared with unknowns from ant nests. In some cases, online images were helpful. Finally, we planted some seeds in order to grow them to an identifiable size. Through these multiple inputs, we were able to identify 48 of the 58 seeds in *P*. *badius* nests. Some seeds remain unidentified, though some of these can be assigned to family.

Seeds were placed in several aspects onto a glass plate above a neutral background and photographed with a DinoCapture 2.0 digital microscope. The microscope added a scale to each image, and these are included on all figures.

## Results

**Fig 1:**
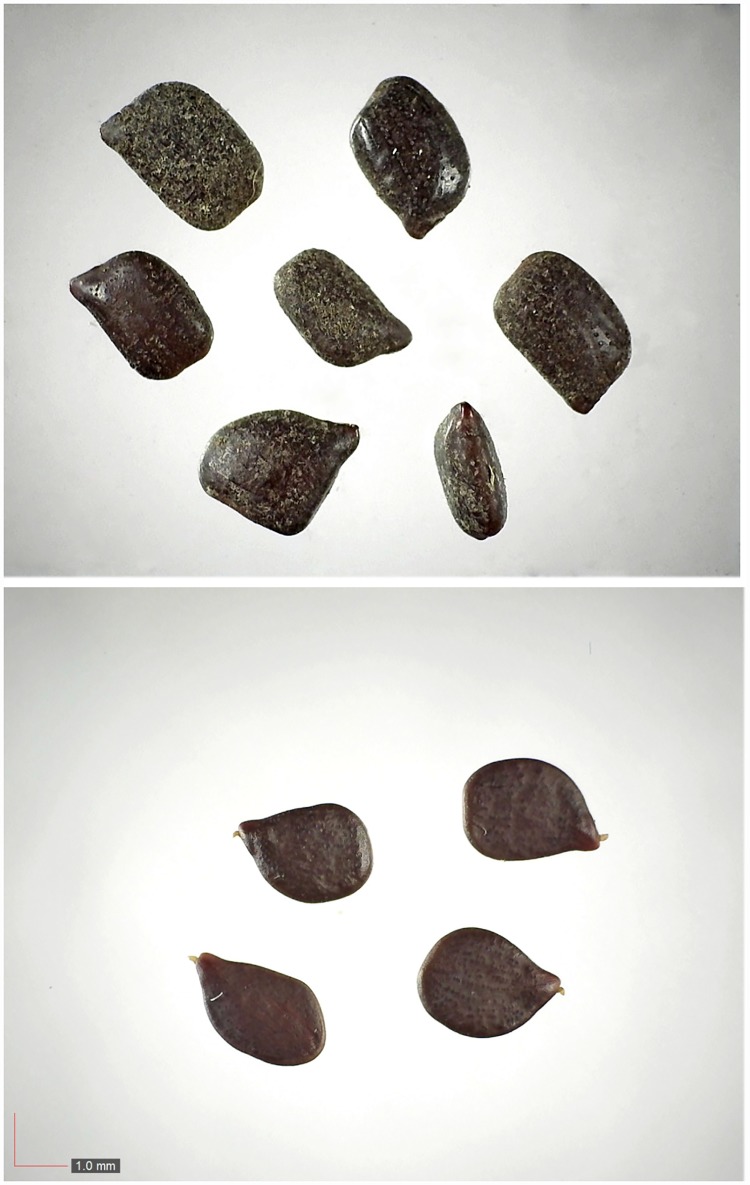
*Chamaecrista nictitans* (Fabaceae). Top: seeds from *P*. *badius* nest; bottom: seeds from herbarium. Table 2 / AOFP / FSU Herbarium

**Fig 2:**
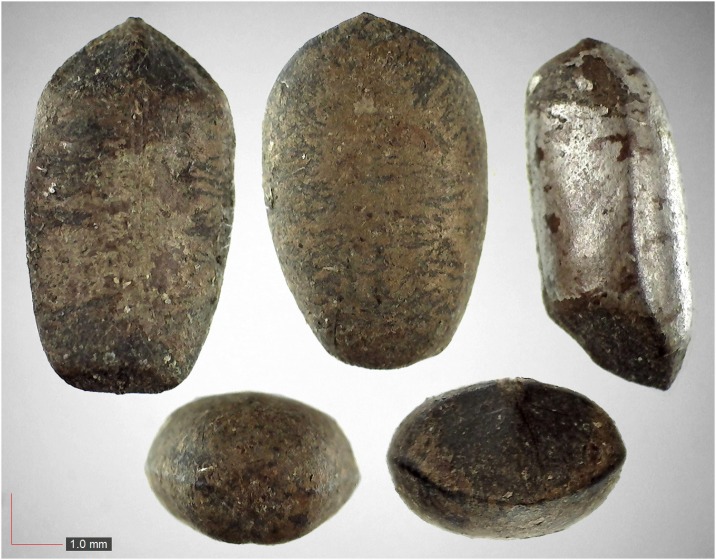
*Cnidoscolus stimulosus* 0.1% (Euphorbiaceae). Table 2 / AOFP / FSU Herbarium

**Fig 3:**
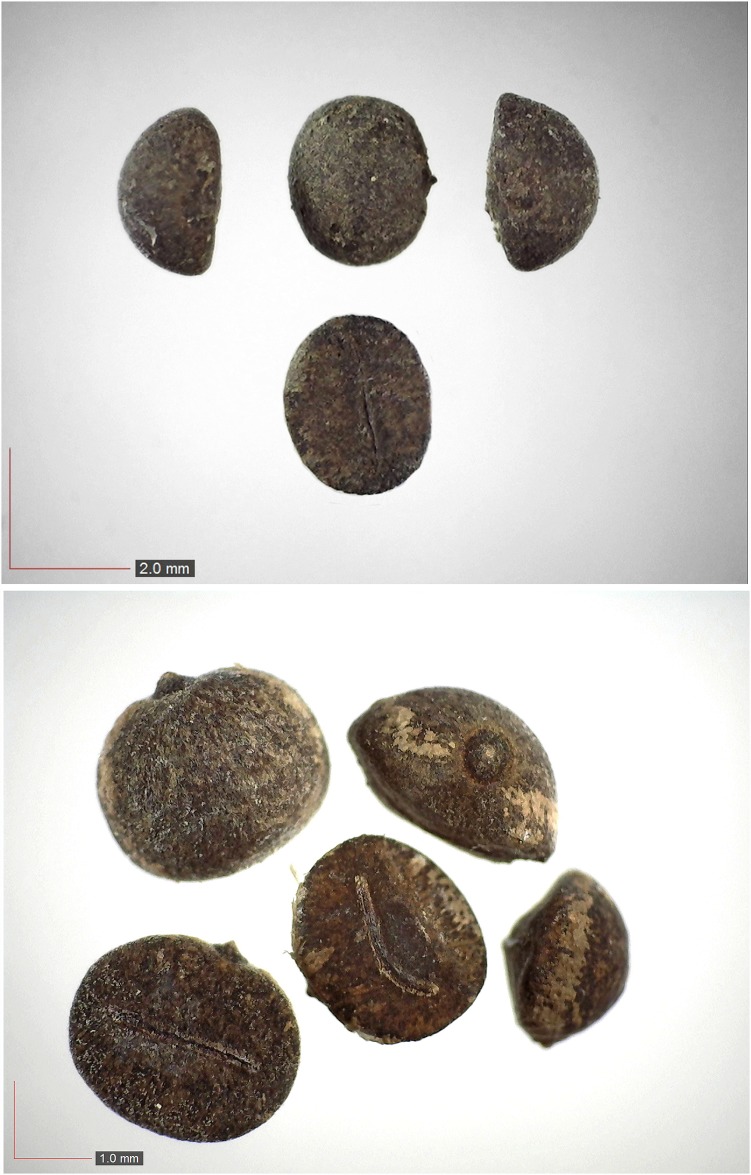
*Commelina erecta* 0.7% (Commelinaceae). Top: seeds from *P*. *badius* nest; bottom: seeds from herbarium. Table 2 / AOFP / FSU Herbarium

**Fig 4:**
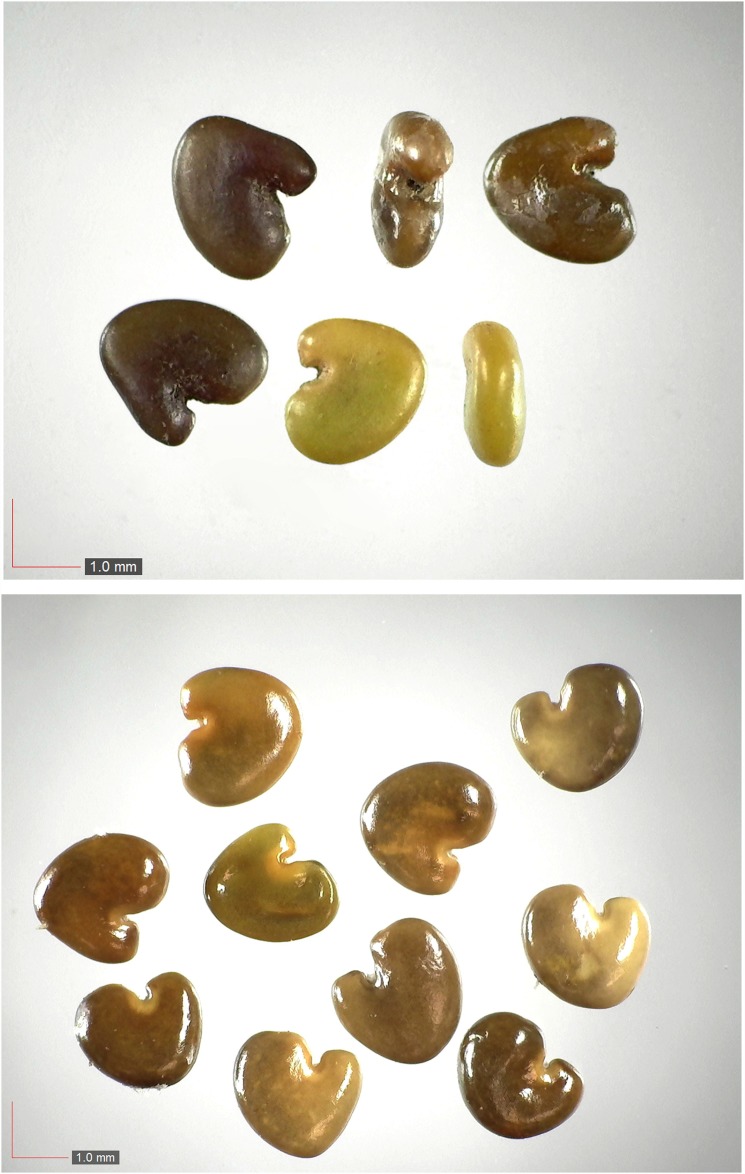
*Crotalaria rotundifolia* 0.1% (Fabaceae). Top: seeds from *P*. *badius* nest; bottom: seeds from herbarium. Table 2 / AOFP / FSU Herbarium

**Fig 5:**
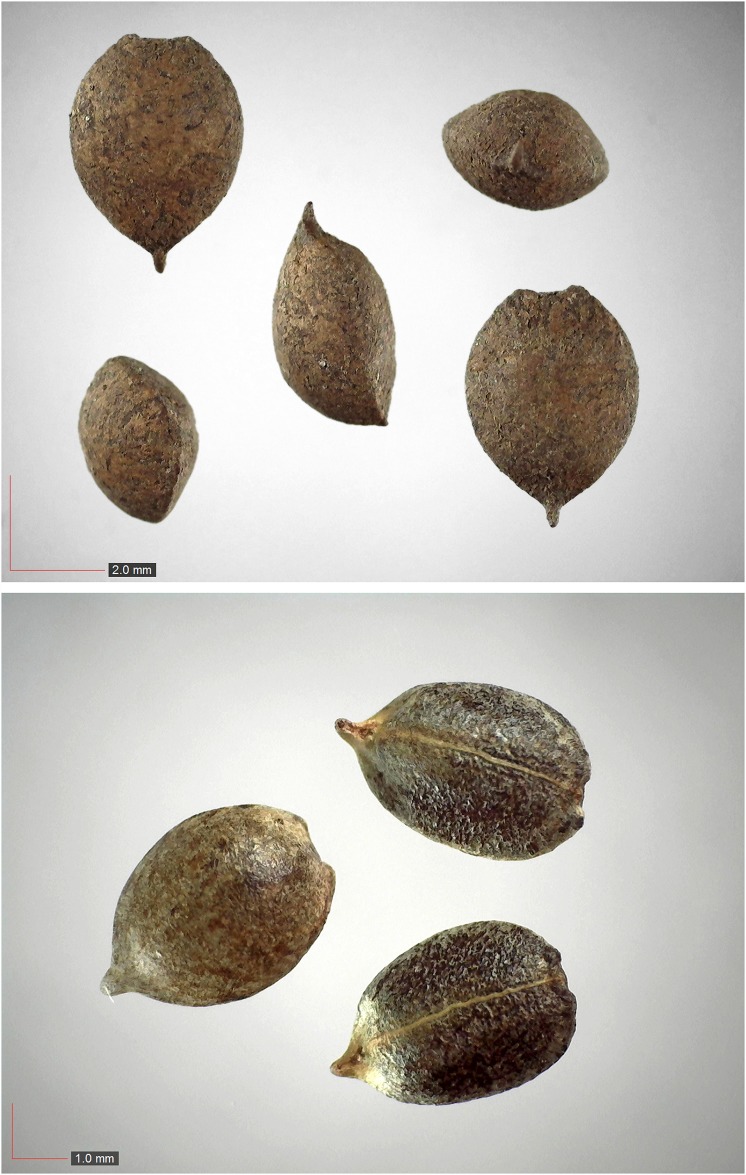
Croton argyranthemus 0.1% (Euphorbiaceae). Top: seeds from *P*. *badius* nest; bottom: seeds from herbarium. Table 2 / AOFP / FSU Herbarium

**Fig 6:**
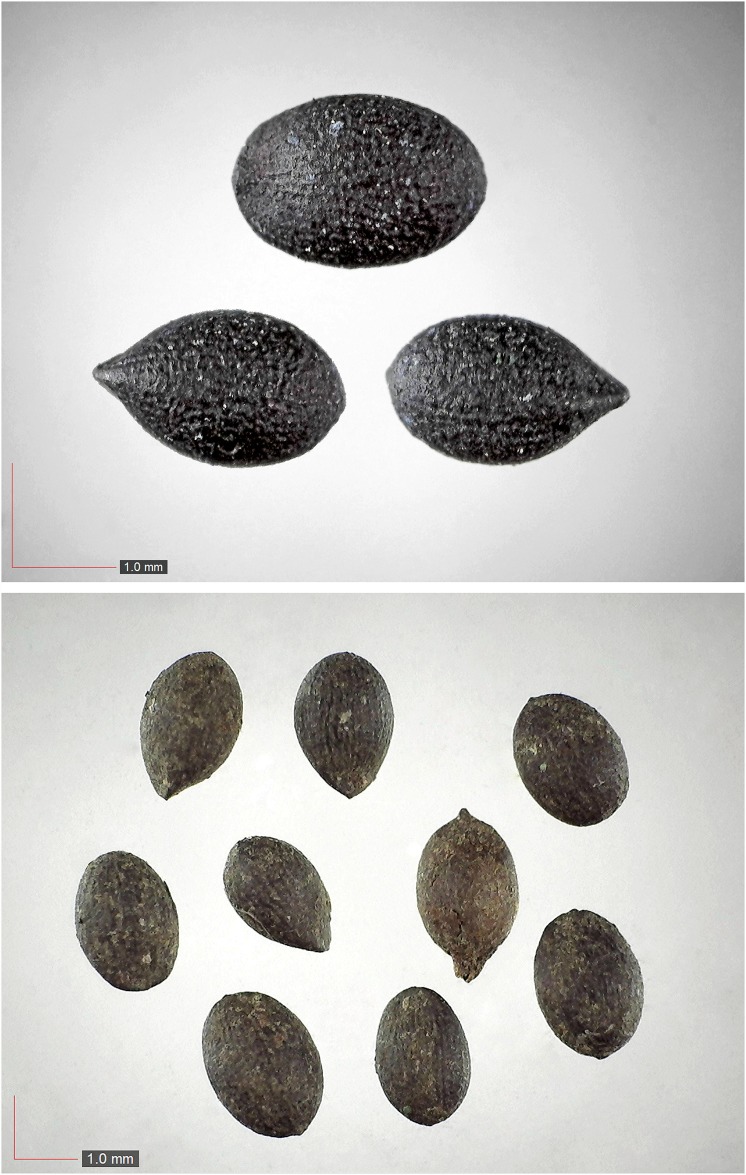
*Croton michauxii* 12.2% (Euphorbiaceae). Seeds from *P*. *badius* nest; Top: close-up shows detail. Table 2 / AOFP / FSU Herbarium

**Fig 7:**
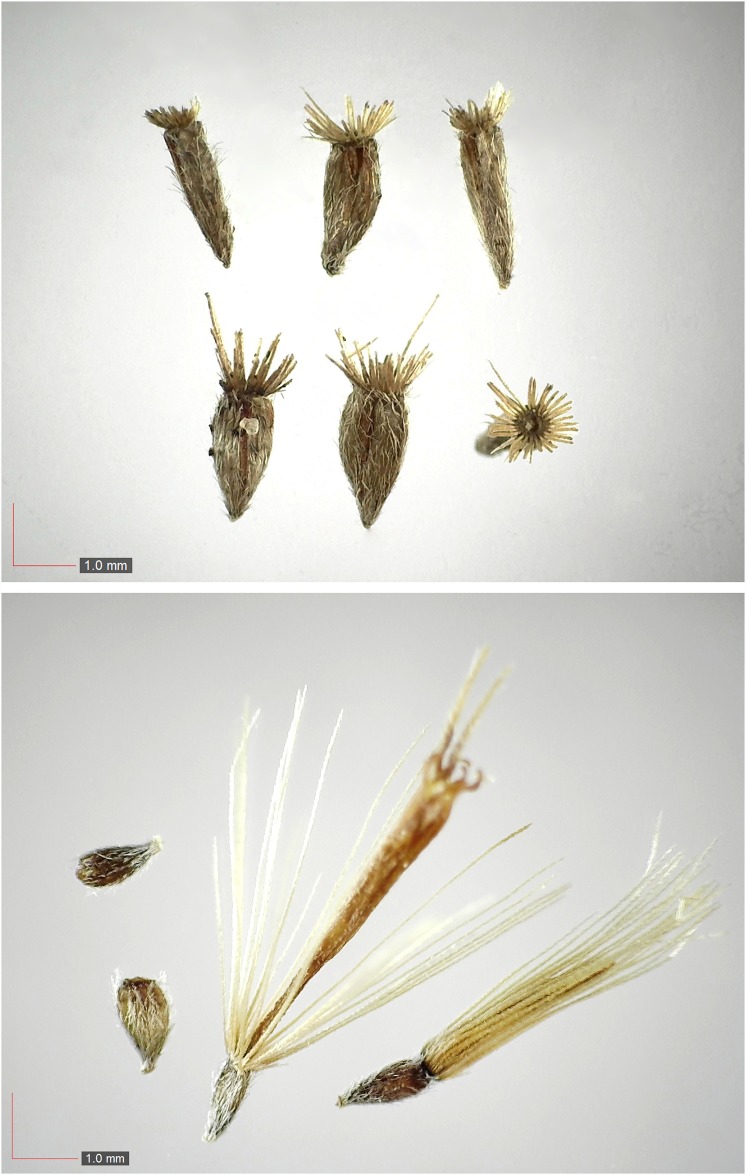
*Chrysopsis lanuginosa* (Asteraceae). Top: seeds from *P*. *badius* nest; bottom: seeds from herbarium. Table 2 / AOFP / FSU Herbarium

**Fig 8:**
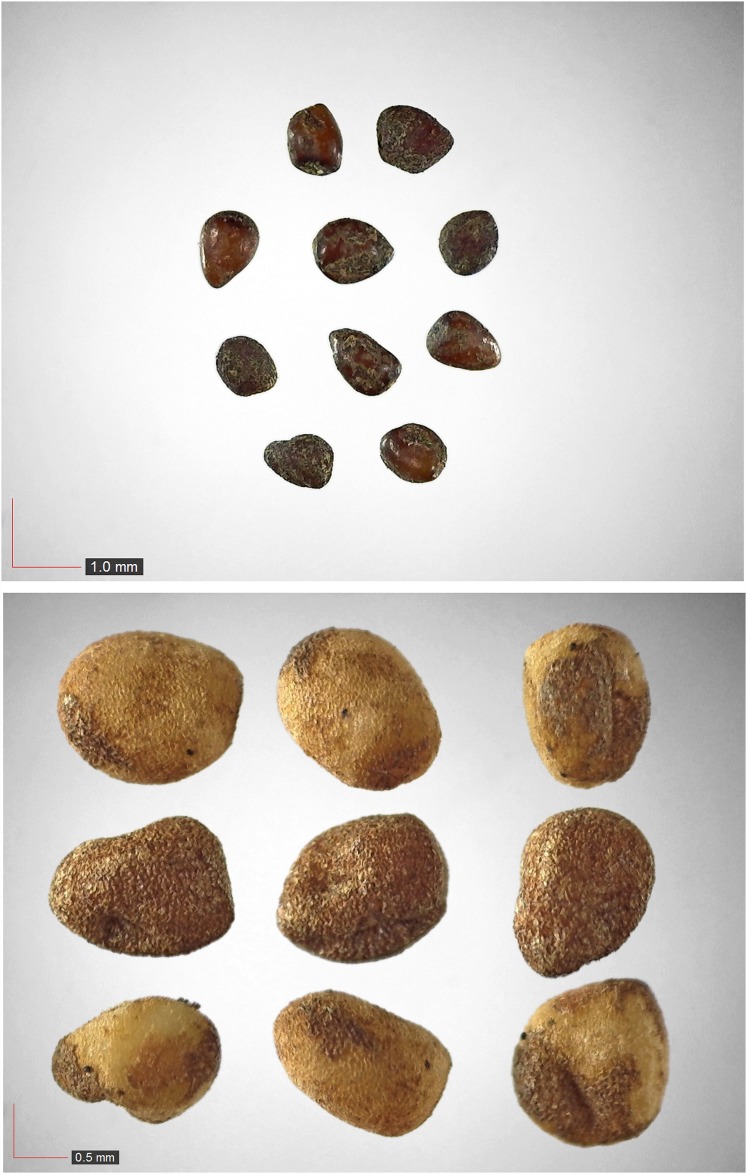
*Cuscuta sp*. (Convulvulaceae). Top: seeds from *P*. *badius* nest; bottom: seeds from herbarium. Table 2 / AOFP / FSU Herbarium

**Fig 9:**
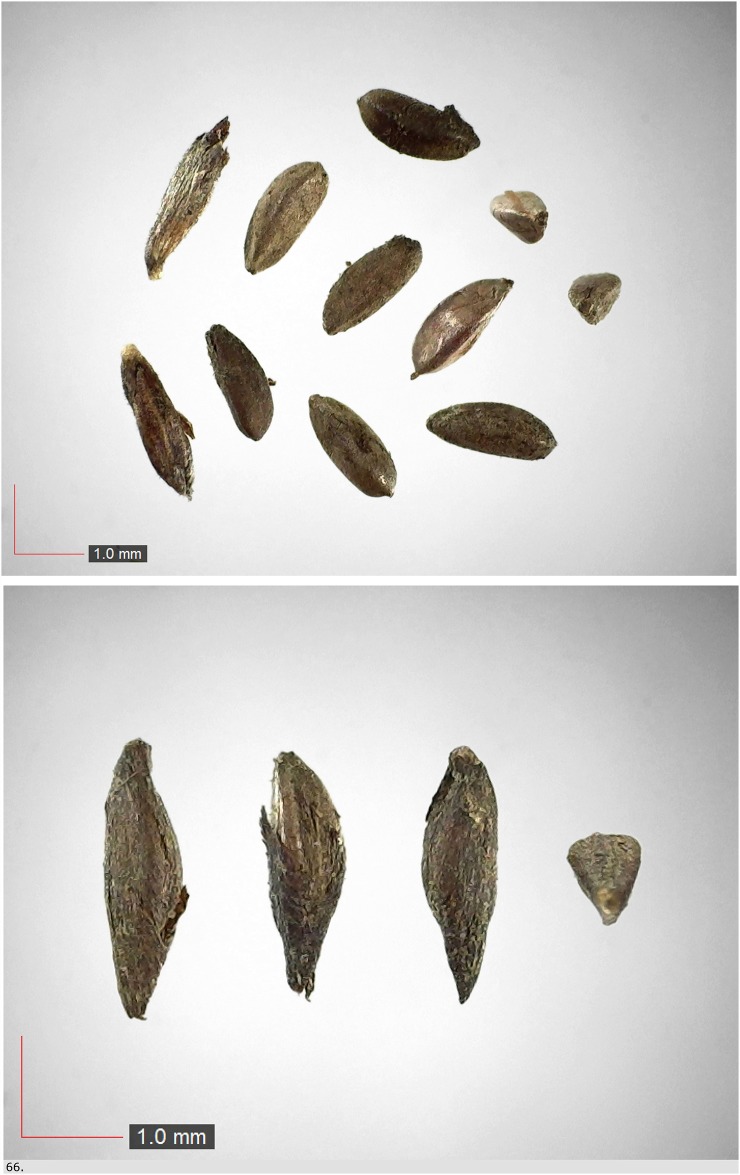
*Cyperus retrorsus* 0.1% (Cyperaceae). Top: seeds from *P*. *badius* nest; bottom: seeds from herbarium. Table 2 / AOFP / FSU Herbarium

**Fig 10:**
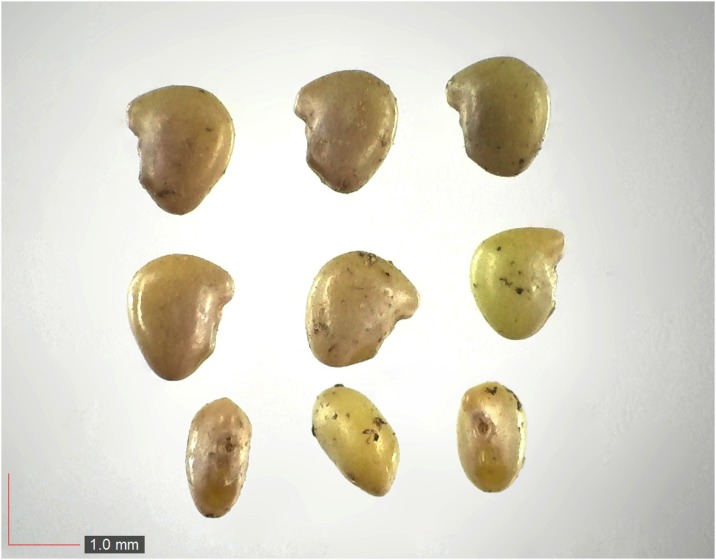
*Dalea pinnata* (Fabaceae). Seeds from *P*. *badius* nest. Table 2 / AOFP / FSU Herbarium

**Fig 11:**
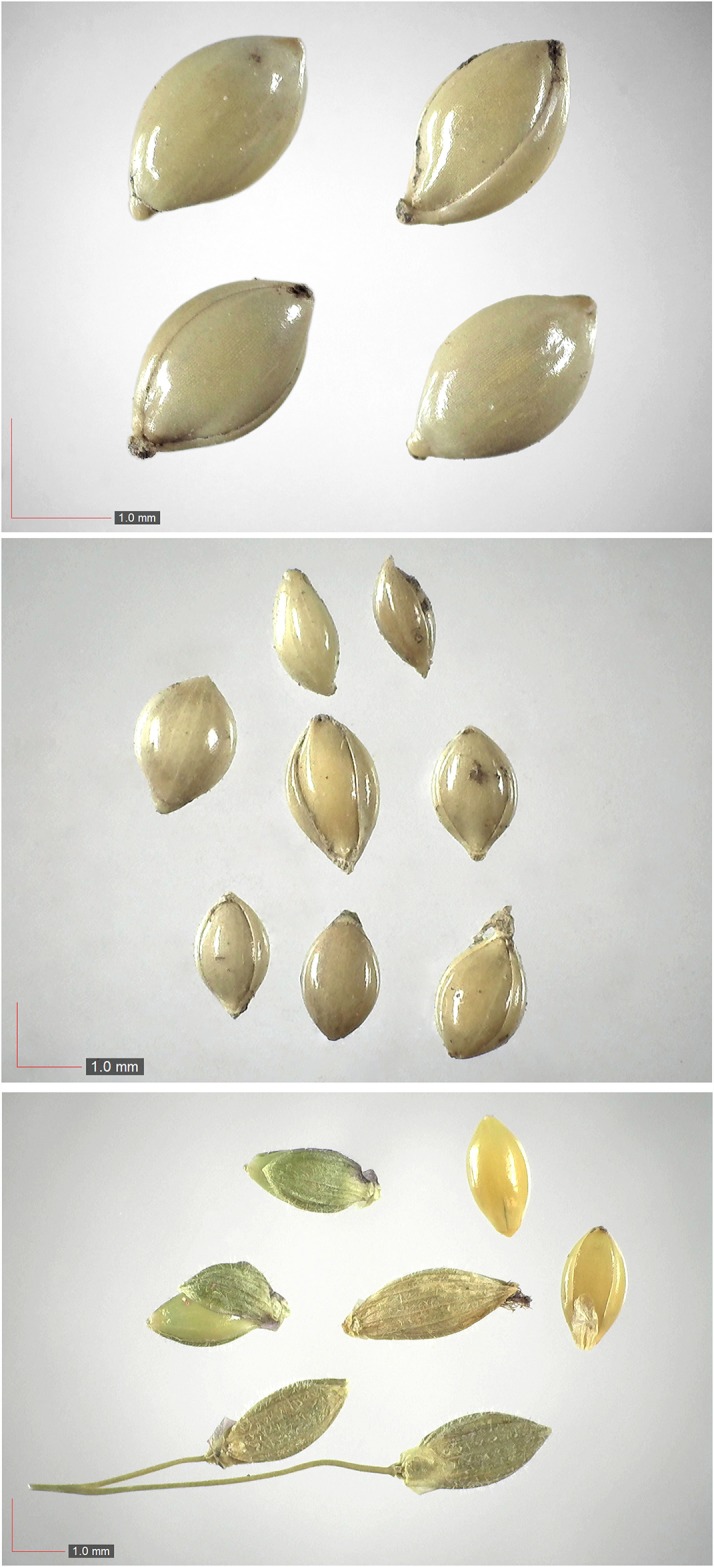
*Dichanthelium commutatum* 35.1% (Poaceae). Top & middle; Seeds from *P*. *badius* nest; Top: close-up shows detail; bottom: seeds from herbarium. Table 2 / AOFP / FSU Herbarium.

**Fig 12:**
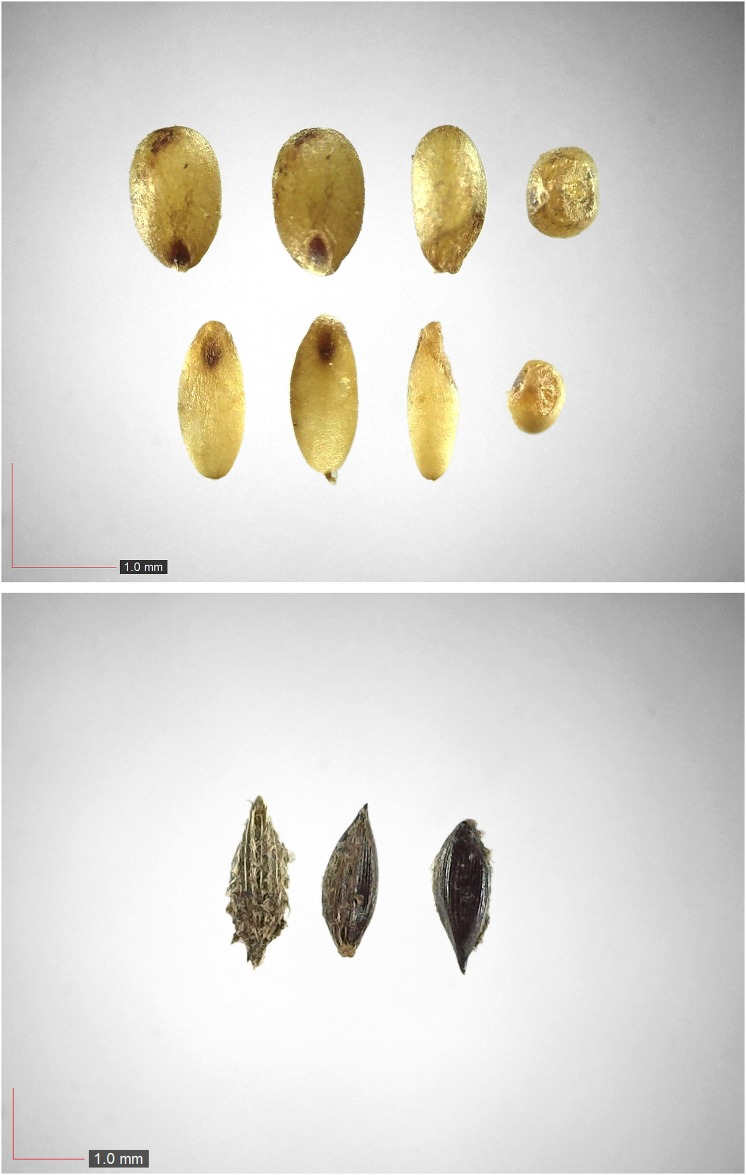
*Digitaria sp*. *A* 5.5% (Poaceae). Upper panel shows immature seeds.Table 2 / AOFP / FSU Herbarium.

**Fig 13:**
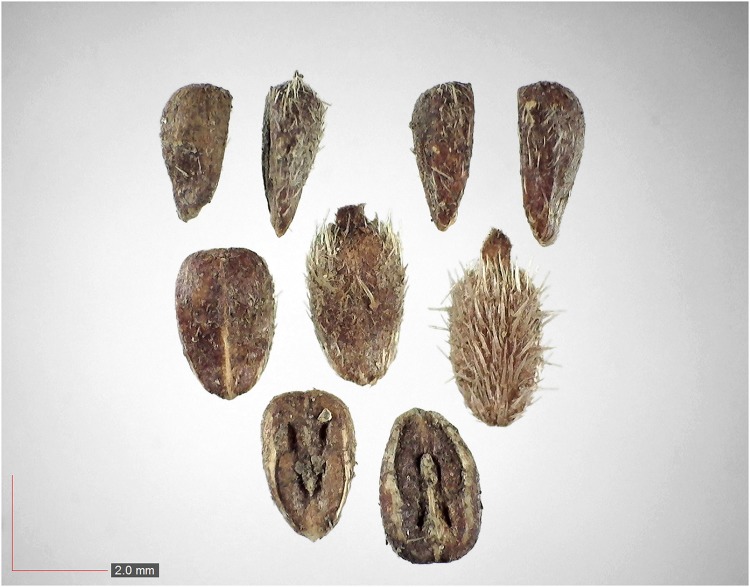
*Diodia teres* 3.2% (Rubiaceae). Table 2 / AOFP / FSU Herbarium

**Fig 14:**
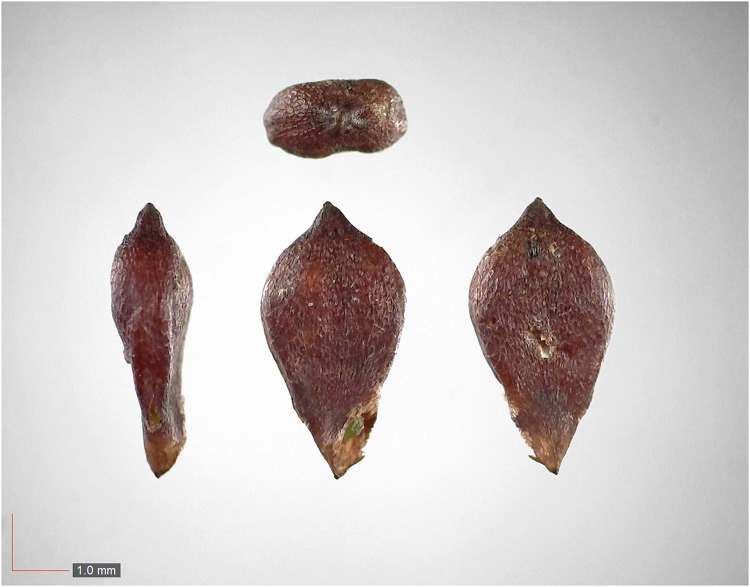
*Eriogonum tomentosum* 0.1% (Polygonaceae). Table 2 / AOFP / FSU Herbarium

**Fig 15:**
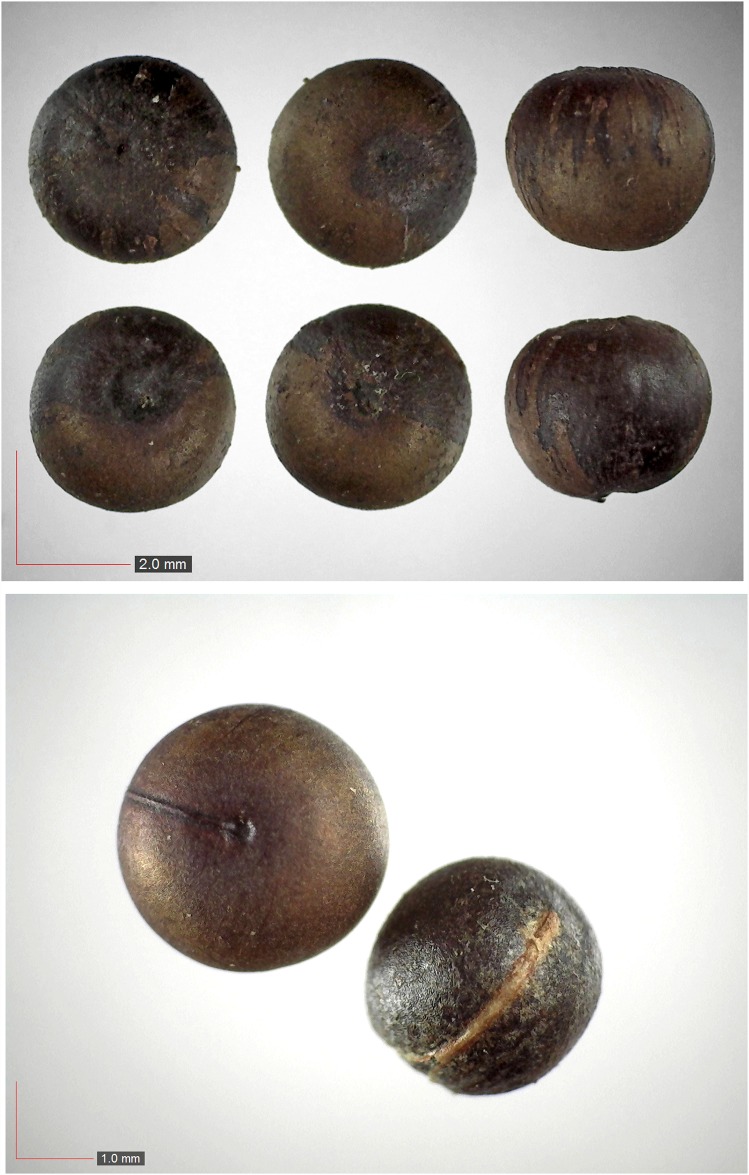
*Euphorbia floridana* (Euphorbiaceae). Top: seeds from *P*. *badius* nest; bottom: seeds from herbarium. Table 2 / AOFP / FSU Herbarium

**Fig 16:**
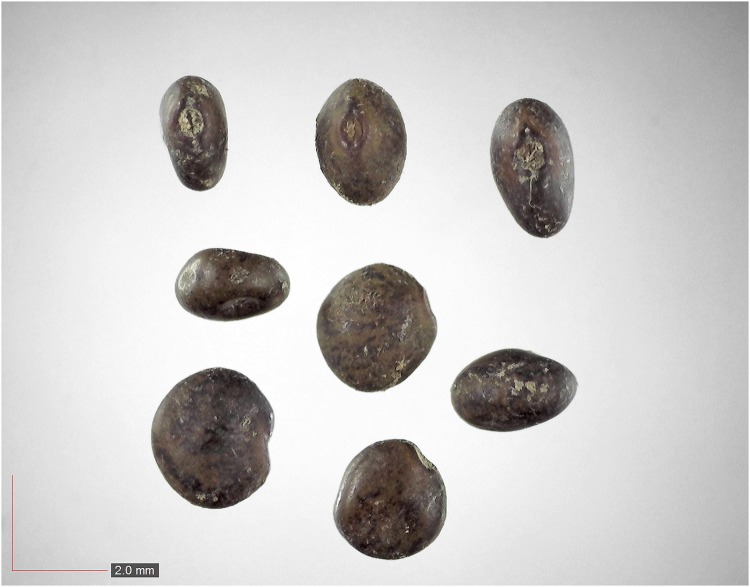
*Galactia sp*. *0*.*3%* (Fabaceae). Table 2 / AOFP / FSU Herbarium

**Fig 17:**
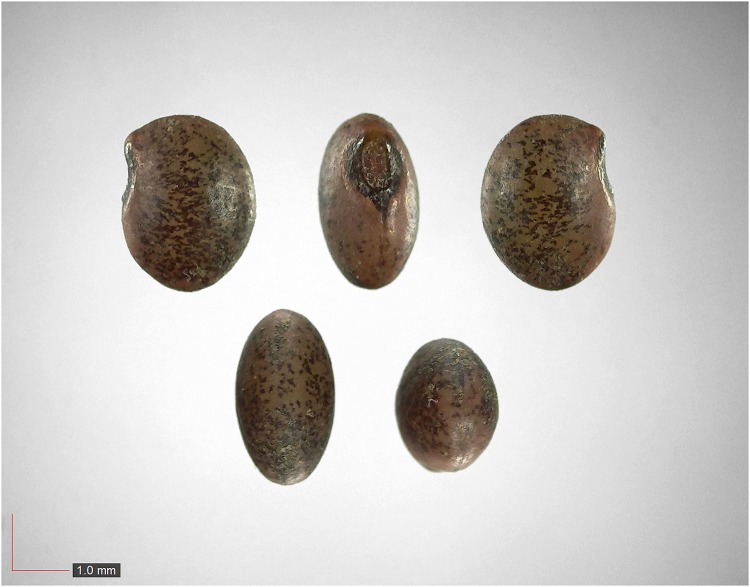
*Galactia volubilis 0*.*1%* (Fabaceae). Table 2 / AOFP / FSU Herbarium

**Fig 18:**
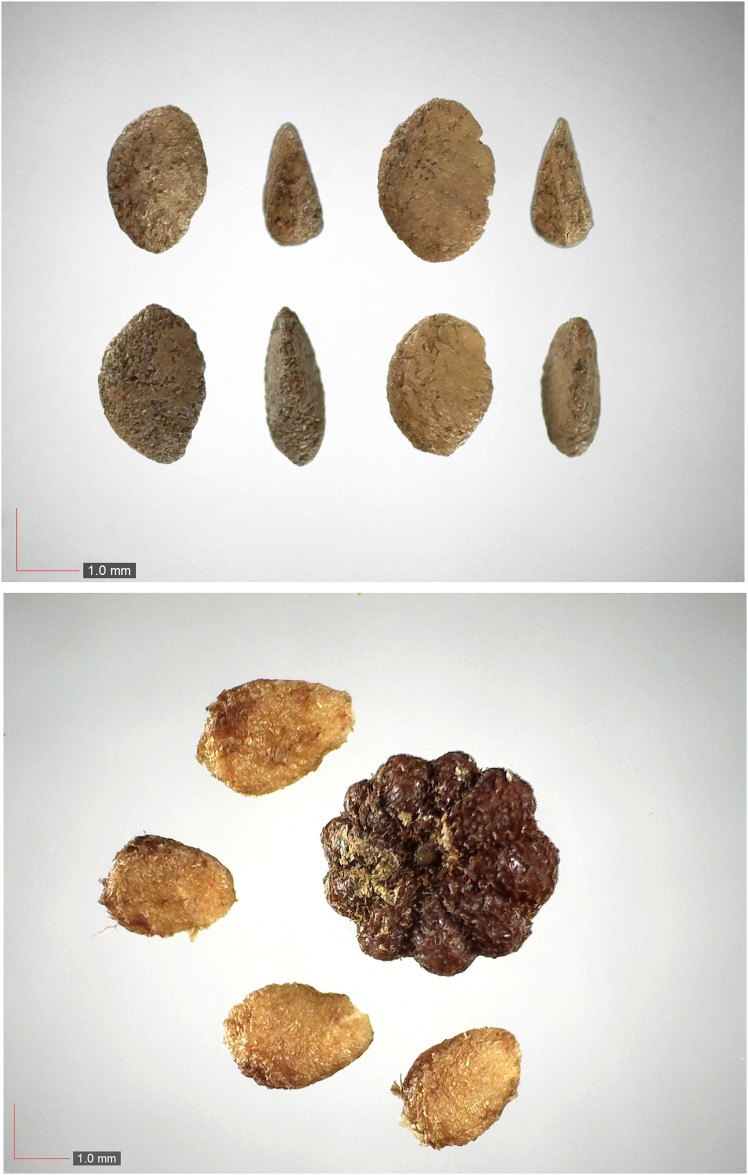
*Gaylussacia dumosa* 0.2% (Ericaceae). Top: seeds from *P*. *badius* nest; bottom: seeds from herbarium. Table 2 / AOFP / FSU Herbarium

**Fig 19:**
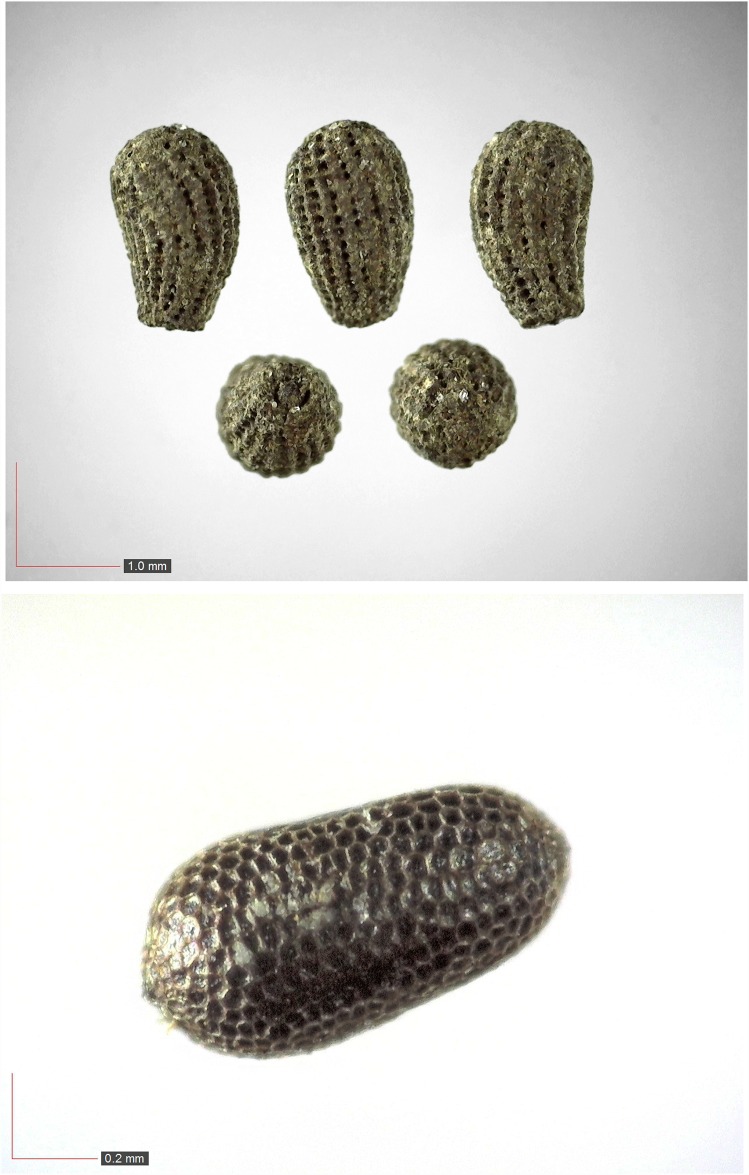
*Hypericum hypericoides* (Clustaceae). Top: seeds from *P*. *badius* nest; bottom: seeds from herbarium. Table 2 / AOFP / FSU Herbarium

**Fig 20:**
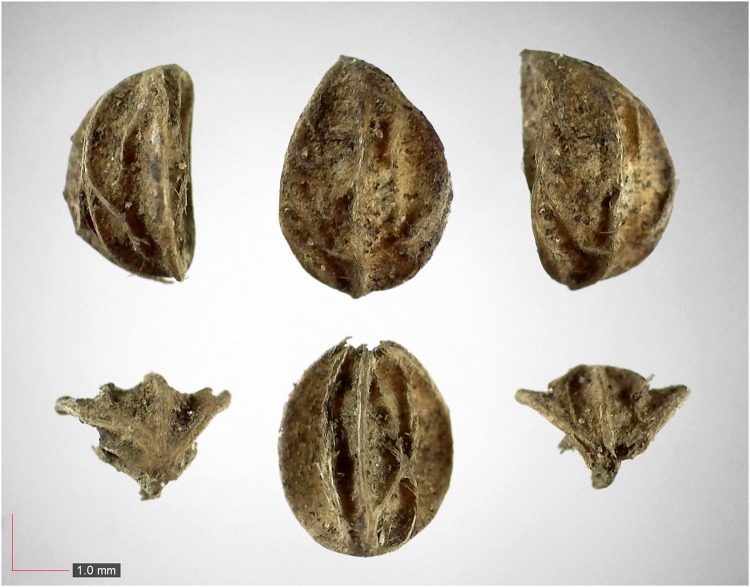
*Ilex myrtifolia* (Ericaceae). Table 2 / AOFP / FSU Herbarium.

**Fig 21:**
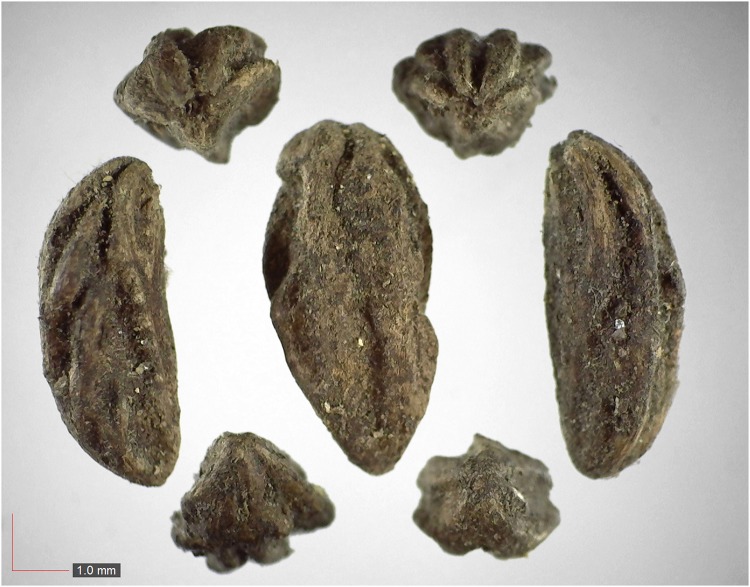
*Ilex sp*. (Ericaceae). Table 2 / AOFP / FSU Herbarium.

Of the 58 types of seeds that were readily separated in the 1989 sample, we identified 48 with confidence. Ten species remain unidentified, but none occurred at greater than 0.1% of the total. All seeds are listed in order of their 1989 frequency in *P*. *badius* nests in Table 1, along with their proportion of the total number of seeds in the 1989 sample, and a hyperlink to the image of each seed in this paper. A second column reports the frequency of the seeds in the 2014–2015 sample. The table also presents the weight of each seed relative to the smallest seeds found in *P*. *badius* nests. Figs 1 to 58 present images of the seeds from *P*. *badius* nests in several aspects, along with two links to online images of the parent plant, one to the Atlas of Florida Plants (AOFP) and one to the Florida State Herbarium specimen images (FSU Herbarium). Figs 1–58 are in alphabetical order. For visual ease, Table 2 presents the seed names in alphabetical order.

**Table 1:** Seed species found in the nests of the Florida harvester ant, *Pogonomyrmex badius*. Each species is linked to its image below. Seeds were collected from 31 nests in 1989 and 9 in 2014. The frequency of each is shown in the columns as percent of total number of seeds. Each seed image has a link to return to this table, a link to the Atlas of Florida Plants at the University of South Florida (AOFP) and a link to the images of the plant specimens in the R.K. Godfrey Herbarium at Florida State University (FSU herbarium).

Plant ID	Family	Fig No.	Abundance 1989 (number %)	Abundance 2014 (number %)	Relative Weight (to smallest)
***Dicanthelium commutatum***	Poaceae	Fig 11	35.10%	11.6%	2.2
***Paspalum setaceum***	Poaceae	Fig 27	29.00%	21.6%	
***Croton michauxii***	Ephorbiaceae	Fig 6	12.20%	37.9%	7.4
***Digitaria sp***. ***A***	Poaceae	Fig 12	5.50%	<0.1%	
***Rhus glabra***	Anacardiaceae	Fig 35	3.70%	0.40%	20
***Trichostema dichotomum***	Lamiaceae	Fig 46	2.60%	<0.1%	4.6
***Diodia teres***	Rubiaceae	Fig 13	2.50%	14.5%	10.7
***Polygonella gracilis***	Polygonaceae	Fig 33	2.40%	0.1%	1.0
***Lespedeza hirta***	Fabaceae	Fig 22	1.70%	0.7%	11
***Commelina erecta***	Commelinaceae	Fig 3	0.70%	<0.1%	23
***Vicia (*****prob.** ***sativa) sp***.	Fabaceae	Fig 47	0.50%	0.6%	37
***Rubus trivialis***	Rosaceae	Fig 36	0.50%	2.3%	3.8
***Vitis rotundifolia***	Vitaceae	Fig 48	0.50%	<0.1%	
***Unidentified 6***	prob. Fabaceae	Fig 54	0.40%	0.70%	
***Opuntia humifusa***	Cactaceae	Fig 26	0.30%	<0.1%	87
***Galactia sp***.	Fabaceae	Fig 16	0.30%	<0.1%	19
***Gaylussacia dumosa***	Ericaceae	Fig 18	0.20%	<0.1%	4.5
***Paspalum notatum***	Poaceae	Fig 28	0.20%	<0.1%	5.3
***Rhus copallinum***	Anacardiaceae	Fig 34	0.20%	3.20%	21
***Stylosanthes biflora***	Fabaceae	Fig 44	0.20%	<0.1%	
***Tradescantia ohiensis***	Commelinaceae	Fig 45	0.20%	<0.1%	
***Cnidoscolus stimulosus***	Euphorbiaceae	Fig 2	0.10%	0.20%	78
***Crotalaria rotundifolia***	Fabaceae	Fig 4	0.10%	0.90%	
***Croton argyranthemus***	Ephorbiaceae	Fig 5	0.10%	<0.1%	40
***Cyperus retrorsus***	Cyperaceae	Fig 9	0.10%	<0.1%	1.0
***Eriogonum tomentosum***	Polygonaceae	Fig 14	0.10%	<0.1%	
***Galactia volubilis***	Fabaceae	Fig 17	0.10%	<0.1%	23
***Stylisma humistrata***	Convulvulaceae	Fig 43	0.10%	0.40%	22
***Smilax auriculata***	Smilacaceae	Fig 41	0.10%	0.10%	77
***Cuscuta sp***.	Convulvulaceae	Fig 8	0.10%	<0.1%	71
***Stillingia sylvatica***	Euphorbiaceae	Fig 42	<0.1%	0.10%	71
***Chamaecrista nictitans***	Fabaceae	Fig 1	<0.1%	<0.1%	
***Chrysopsis lanuginosa***	Asteraceae	Fig 7	<0.1%	<0.1%	1.8
***Dalea pinnata***	Fabaceae	Fig 10	<0.1%	<0.1%	4.3
***Digitaria sp***. ***B***	Poaceae	Fig 12	<0.1%	<0.1%	
***Euphorbia floridana***	Ephorbiaceae	Fig 15	<0.1%	<0.1%	47
***Hypericum hypericoides***	Clustaceae	Fig 19	<0.1%	<0.1%	
***Ilex myrtifolia***	Ericaeae	Fig 20	<0.1%	<0.1%	
***Ilex sp***.	Ericaeae	Fig 21	<0.1%	<0.1%	21
***Magnolia grandiflora***	Magoliaceae	Fig 23	<0.1%	<0.1%	
***Magnolia virginiana***	Magoliaceae	Fig 24	<0.1%	<0.1%	
***Nyssa sylvatica***	Cornaceae	Fig 25	<0.1%	<0.1%	
***Phytolacca americiana***	Phytolaccaceae	Fig 29	<0.1%	<0.1%	15
***Pinus ellliottii***	Pinaceae	Fig 30	<0.1%	0.10%	40
***Pinus palustris***	Pinaceae	Fig 31	<0.1%	<0.1%	30
***Pinus taeda***	Pinaceae	Fig 32	<0.1%	<0.1%	
***Rumex hastatulus***	Polygonaceae	Fig 37	<0.1%	<0.1%	
***Scleria sp***. ***A***	Cyperaceae	Fig 38	<0.1%	<0.1%	17
***Scleria sp***. ***B***	Cyperaceae	Fig 39	<0.1%	<0.1%	18
***Senna obtusifolia***	Fabaceae	Fig 40	<0.1%	<0.1%	
***Unidentified 1***		Fig 49	<0.1%	0.10%	
***Unidentified 2***		Fig 50	<0.1%	<0.1%	
***Unidentified 3***		Fig 51	<0.1%	<0.1%	
***Unidentified 4***		Fig 52	<0.1%	<0.1%	
***Unidentified 5***		Fig 53	<0.1%	<0.1%	
***Unidentified 7***		Fig 55	<0.1%	<0.1%	
***Unidentified 8***		Fig 56	<0.1%	<0.1%	
***Unidentified 9***		Fig 57	<0.1%	<0.1%	
***Unidentified 10***		Fig 58	<0.1%	<0.1%	
**Other**			n/a	4.60%	

**Table 2:** The species in Table 1 arranged alphabetically.

Plant ID	Family	Fig No.	Abundance 1989 (number %)	Abundance 2014 (number %)	Relative Weight (to smallest)
***Chamaecrista nictitans***	Fabaceae	Fig 1	<0.1%	<0.1%	
***Chrysopsis lanuginosa***	Asteraceae	Fig 7	<0.1%	<0.1%	1.8
***Cnidoscolus stimulosus***	Euphorbiaceae	Fig 2	0.10%	0.20%	78
***Commelina erecta***	Commelinaceae	Fig 3	0.70%	<0.1%	23
***Crotalaria rotundifolia***	Fabaceae	Fig 4	0.10%	0.90%	
***Croton argyranthemus***	Ephorbiaceae	Fig 5	0.10%	<0.1%	40
***Croton michauxii***	Ephorbiaceae	Fig 6	12.20%	37.9%	7.4
***Cuscuta sp***.	Convulvulaceae	Fig 8	0.10%	<0.1%	71
***Cyperus retrorsus***	Cyperaceae	Fig 9	0.10%	<0.1%	1.0
***Dalea pinnata***	Fabaceae	Fig 10	<0.1%	<0.1%	4.3
***Dicanthelium commutatum***	Poaceae	Fig 11	35.10%	11.6%	2.2
***Digitaria sp A***.	Poaceae	Fig 12	5.50%	<0.1%	
***Digitaria sp B***.	Poaceae	Fig 52	<0.1%	<0.1%	
***Diodia teres***	Rubiaceae	Fig 13	2.50%	14.5%	10.7
***Eriogonum tomentosum***	Polygonaceae	Fig 14	0.10%	<0.1%	
***Euphorbia floridana***	Ephorbiaceae	Fig 15	<0.1%	<0.1%	47
***Galactia sp***.	Fabaceae	Fig 16	0.30%	<0.1%	19
***Galactia volubilis***	Fabaceae	Fig 17	0.10%	<0.1%	23
***Gaylussacia dumosa***	Ericaceae	Fig 18	0.20%	<0.1%	4.5
***Hypericum hypericoides***	Clustaceae	Fig 19	<0.1%	<0.1%	
***Ilex myrtifolia***	Ericaeae	Fig 20	<0.1%	<0.1%	
***Ilex sp***.	Ericaeae	Fig 21	<0.1%	<0.1%	21
***Lespedeza hirta***	Fabaceae	Fig 22	1.70%	0.7%	11
***Nyssa sylvatica***	Cornaceae	Fig 25	<0.1%	<0.1%	
***Opuntia humifusa***	Cactaceae	Fig 26	0.30%	<0.1%	87
***Other***			n/a	4.60%	
***Paspalum notatum***	Poaceae	Fig 28	0.20%	<0.1%	5.3
***Paspalum setaceum***	Poaceae	Fig 27	29.00%	21.6%	
***Phytolacca americana***	Phytolaccaceae	Fig 29	<0.1%	<0.1%	15
***Pinus ellliottii***	Pinaceae	Fig 30	<0.1%	0.10%	40
***Pinus palustris***	Pinaceae	Fig 31	<0.1%	<0.1%	30
***Pinus taeda***	Pinaceae	Fig 32	<0.1%	<0.1%	
***Polygonella gracilis***	Polygonaceae	Fig 33	2.40%	0.1%	1.0
***Rhus copallinum***	Anacardiaceae	Fig 34	0.20%	3.20%	21
***Rhus glabra***	Anacardiaceae	Fig 35	3.70%	0.40%	20
***Rubus trivialis***	Rosaceae	Fig 36	0.50%	2.3%	3.8
***Rumex hastatulus***	Polygonaceae	Fig 37	<0.1%	<0.1%	
***Scleria sp***. ***A***	Cyperaceae	Fig 38	<0.1%	<0.1%	17
***Scleria sp***. ***B***	Cyperaceae	Fig 39	<0.1%	<0.1%	18
***Senna obtusifolia***	Fabaceae	Fig 40	<0.1%	<0.1%	
***Smilax auriculata***	Smilacaceae	Fig 41	0.10%	0.10%	77
***Stillingia sylvatica***	Euphorbiaceae	Fig 42	<0.1%	0.10%	71
***Stylisma humistrata***	Convulvulaceae	Fig 43	0.10%	0.40%	22
***Stylosanthes biflora***	Fabaceae	Fig 44	0.20%	<0.1%	
***Tradescantia ohiensis***	Commelinaceae	Fig 45	0.20%	<0.1%	
***Trichostema dichotomum***	Lamiaceae	Fig 46	2.60%	<0.1%	4.6
***Unidentified 1***		Fig 49	<0.1%	0.10%	
***Unidentified 10***		Fig 58	<0.1%	<0.1%	
***Unidentified 2***		Fig 50	<0.1%	<0.1%	
***Unidentified 3***		Fig 51	<0.1%	<0.1%	
***Unidentified 4***		Fig 52	<0.1%	<0.1%	
***Unidentified 5***		Fig 53	<0.1%	<0.1%	
***Unidentified 6***	prob. Fabaceae	Fig 54	0.40%	0.70%	
***Unidentified 7***		Fig 55	<0.1%	<0.1%	
***Unidentified 8***		Fig 56	<0.1%	<0.1%	
***Unidentified 9***		Fig 57	<0.1%	<0.1%	
***Vicia sp***.	Fabaceae	Fig 47	0.50%	0.6%	37
***Vitis rotundifolia***	Vitaceae	Fig 48	0.50%	<0.1%	

**Fig 22:**
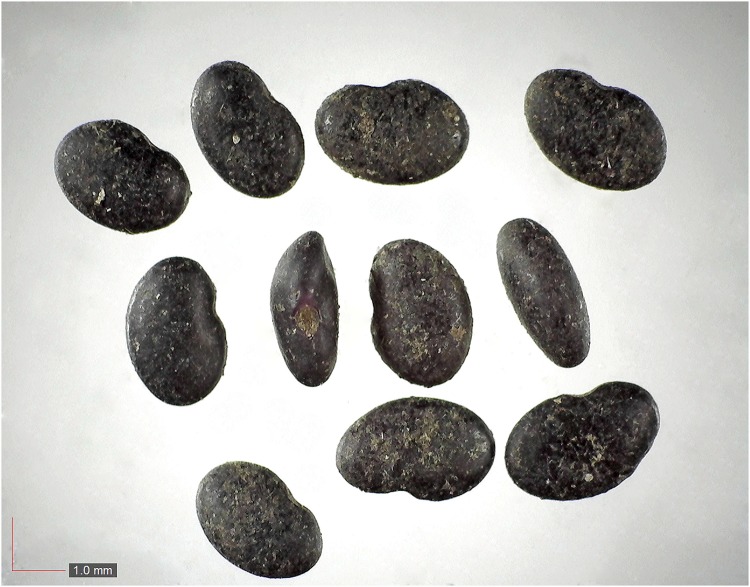
*Lespedeza hirta* 1.7% (Fabaceae). Table 2 / AOFP / FSU Herbarium.

**Fig 23:**
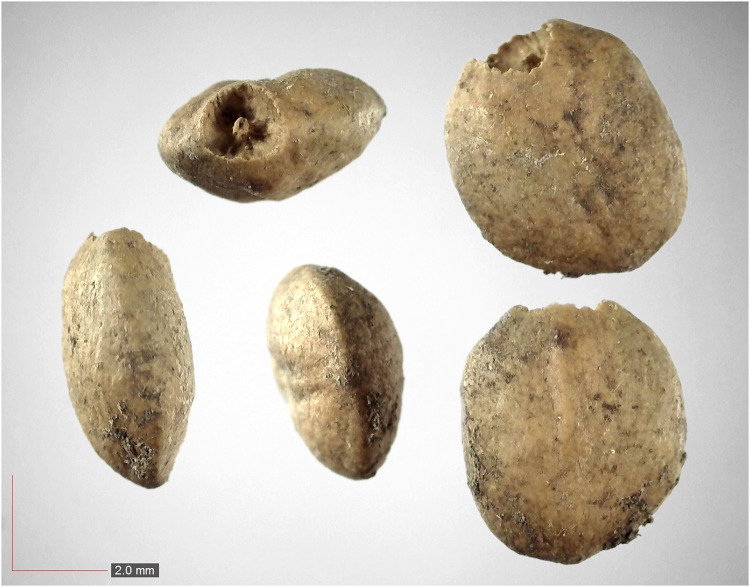
*Magnolia grandiflora* (Magnoliaceae). Table 2 / AOFP / FSU Herbarium.

**Fig 24:**
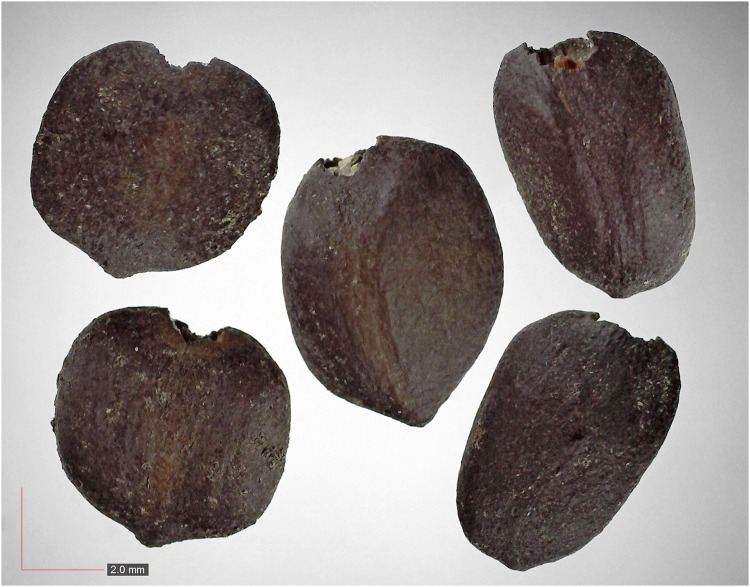
*Magnolia virginiana* (Magnoliaceae). Table 2 / AOFP / FSU Herbarium

**Fig 25:**
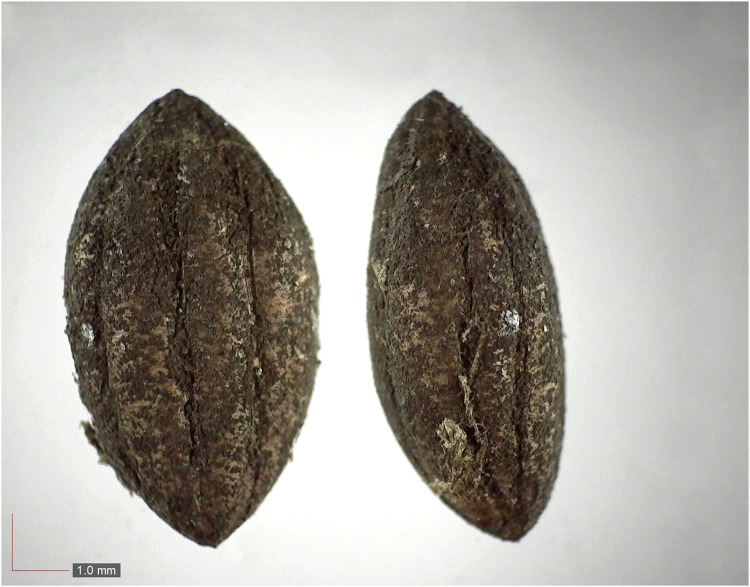
*Nyssa sylvatica* (Cornaceae). Table 2 / AOFP / FSU Herbarium.

**Fig 26:**
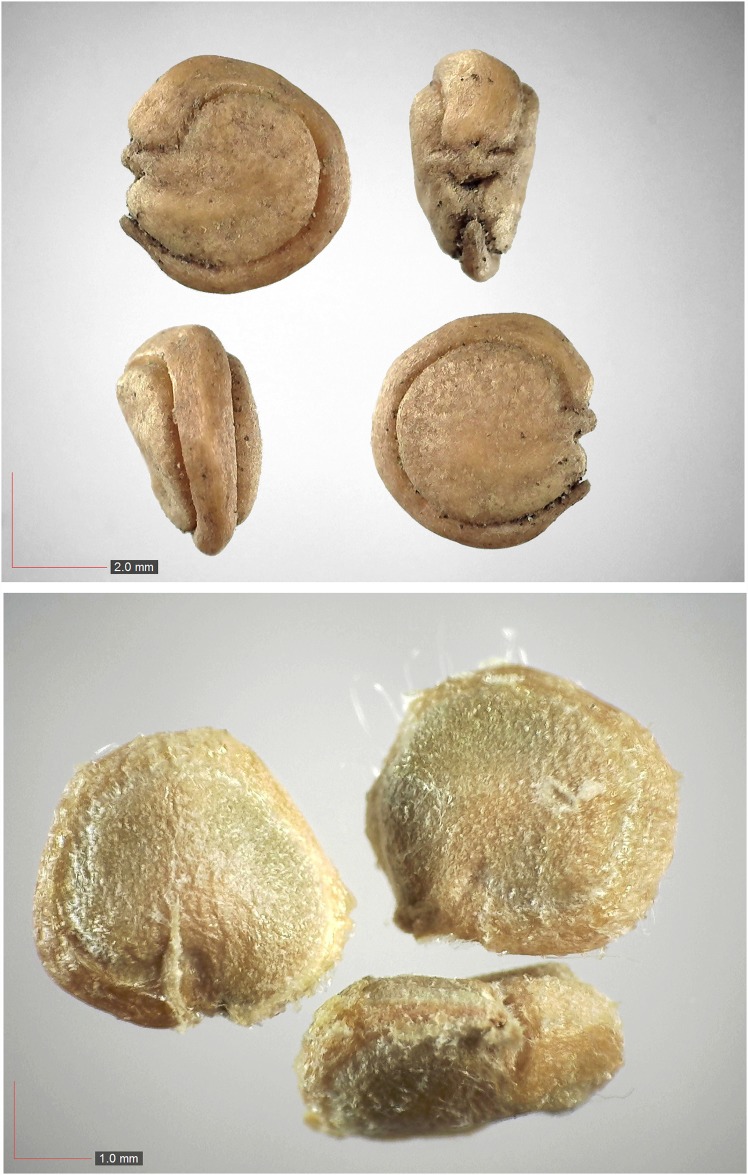
*Opuntia humifusa* (Cactaceae). Top: seeds from *P*. *badius* nest; bottom: seeds from herbarium. Table 2 / AOFP / FSU Herbarium.

**Fig 27:**
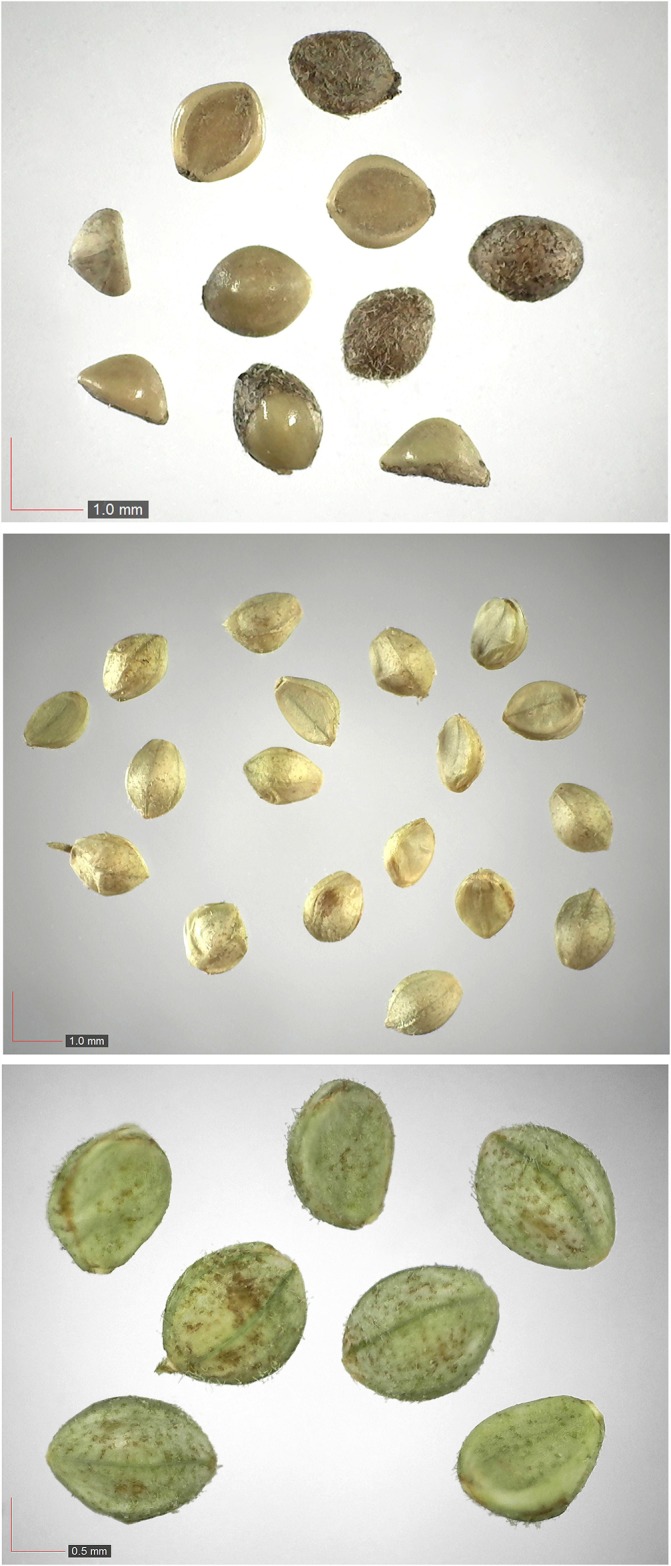
*Paspalum setaceum* 29% Poaceae). Top: seeds from *P*. *badius* nest; middle: seeds from herbarium; bottom: seeds collected from plants in the field. Table 2 / AOFP / FSU Herbarium.

**Fig 28:**
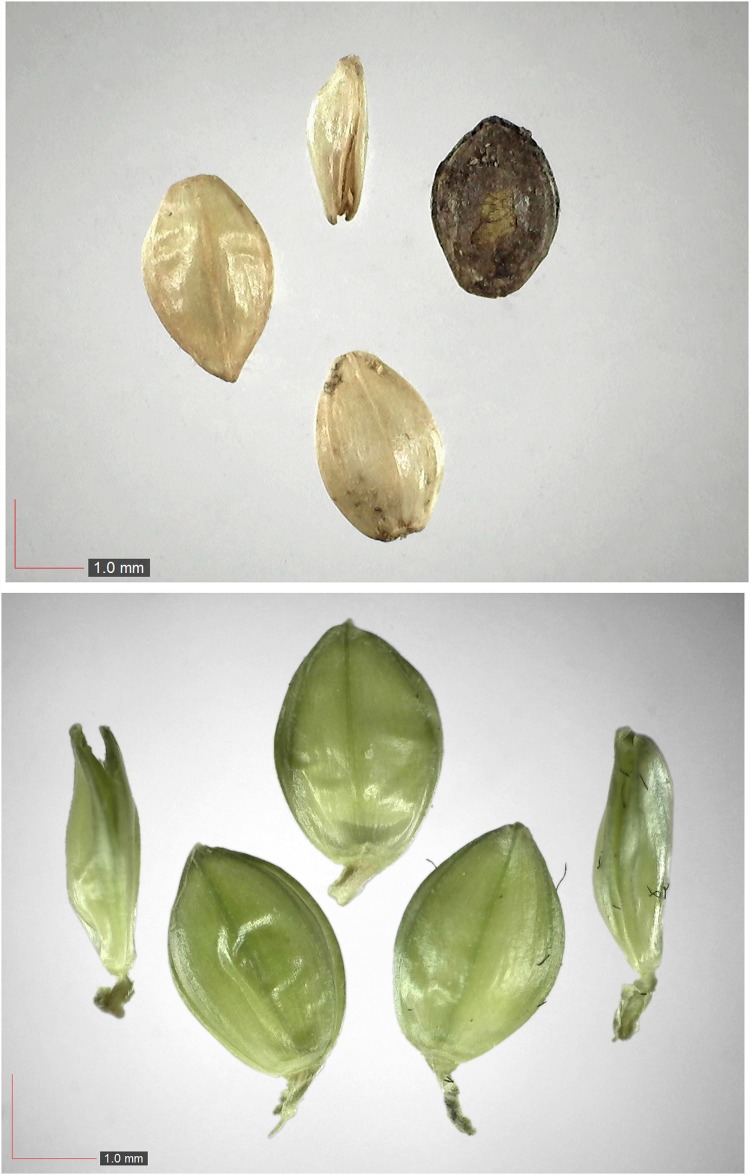
*Paspalum notatum* 0.2% Poaceae). Lower panel: fresh seeds. Table 2 / AOFP / FSU Herbarium.

**Fig 29:**
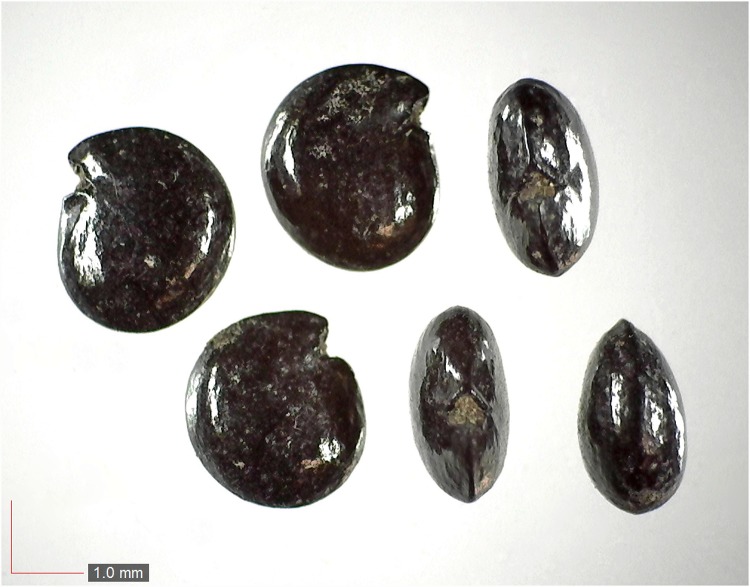
*Phytolacca americana* (Phytolaccaceae). Table 2 / AOFP / FSU Herbarium.

**Fig 30:**
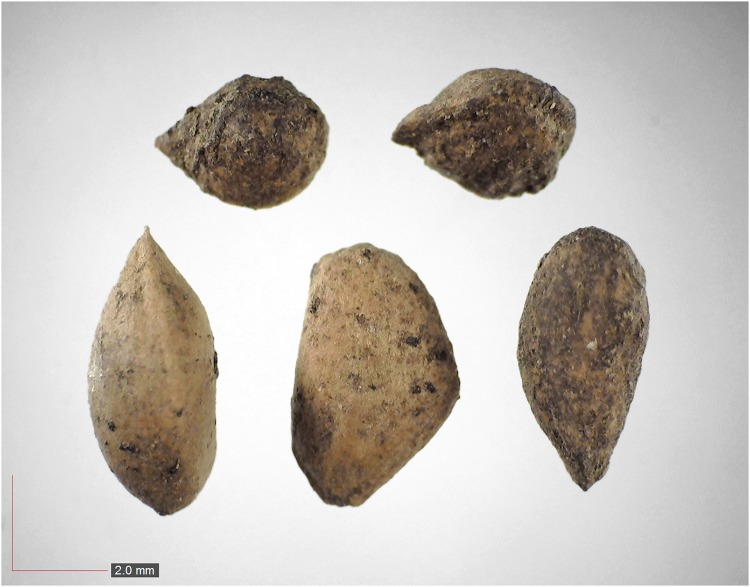
Pinus elliottii (Pinaceae). Table 2 / AOFP / FSU Herbarium.

**Fig 31:**
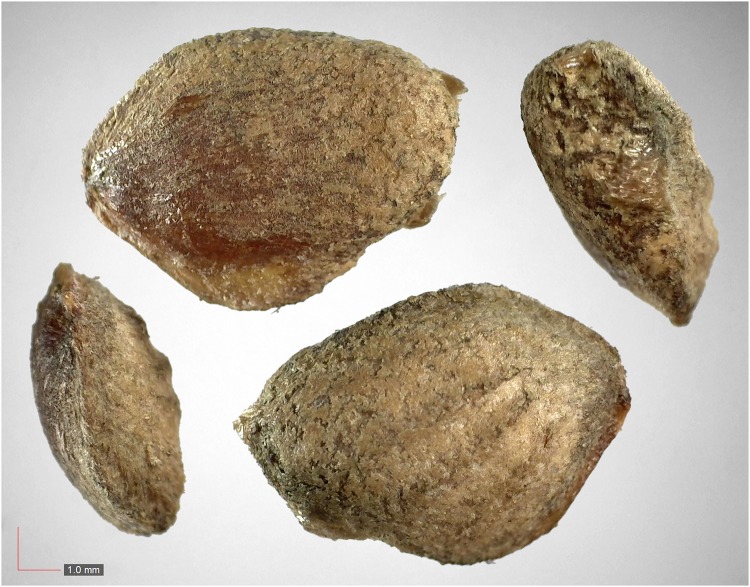
*Pinus palustris* 0.0% (Pinaceae). Table 2 / AOFP / FSU Herbarium.

**Fig 32:**
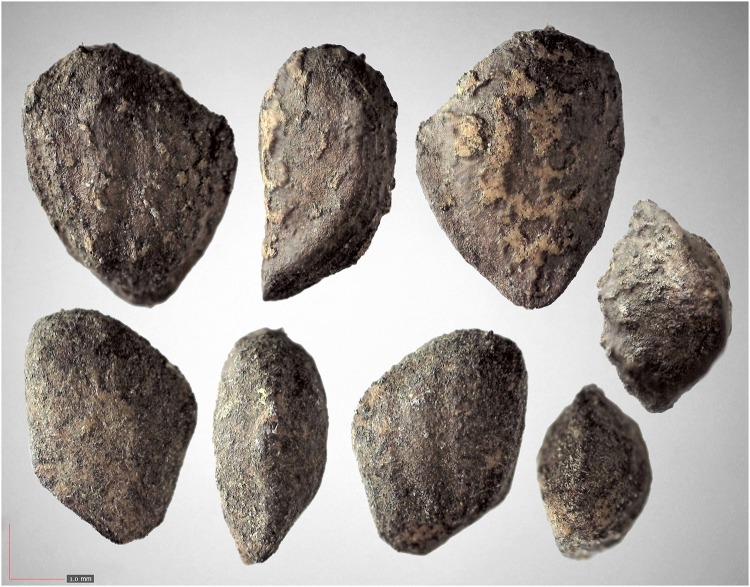
*Pinus taeda* (Pinaceae). Table 2 / AOFP / FSU Herbarium.

**Fig 33:**
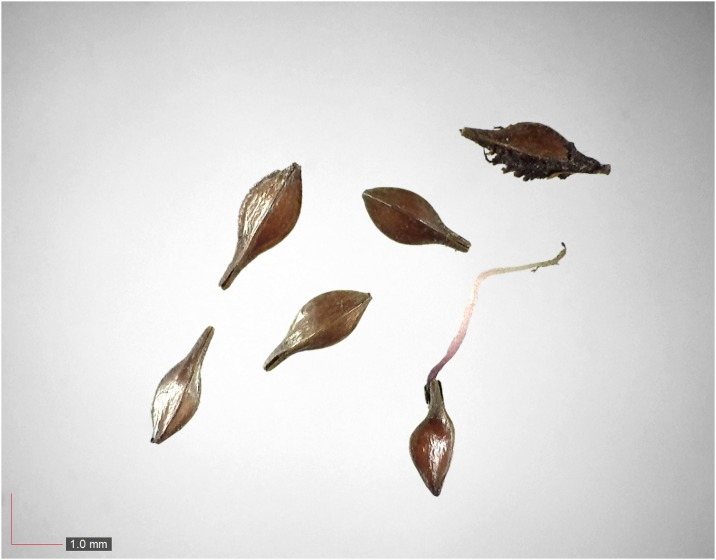
*Polygonella gracilis* 2.4% (Polygonaceae). Table 2 / AOFP / FSU Herbarium.

**Fig 34:**
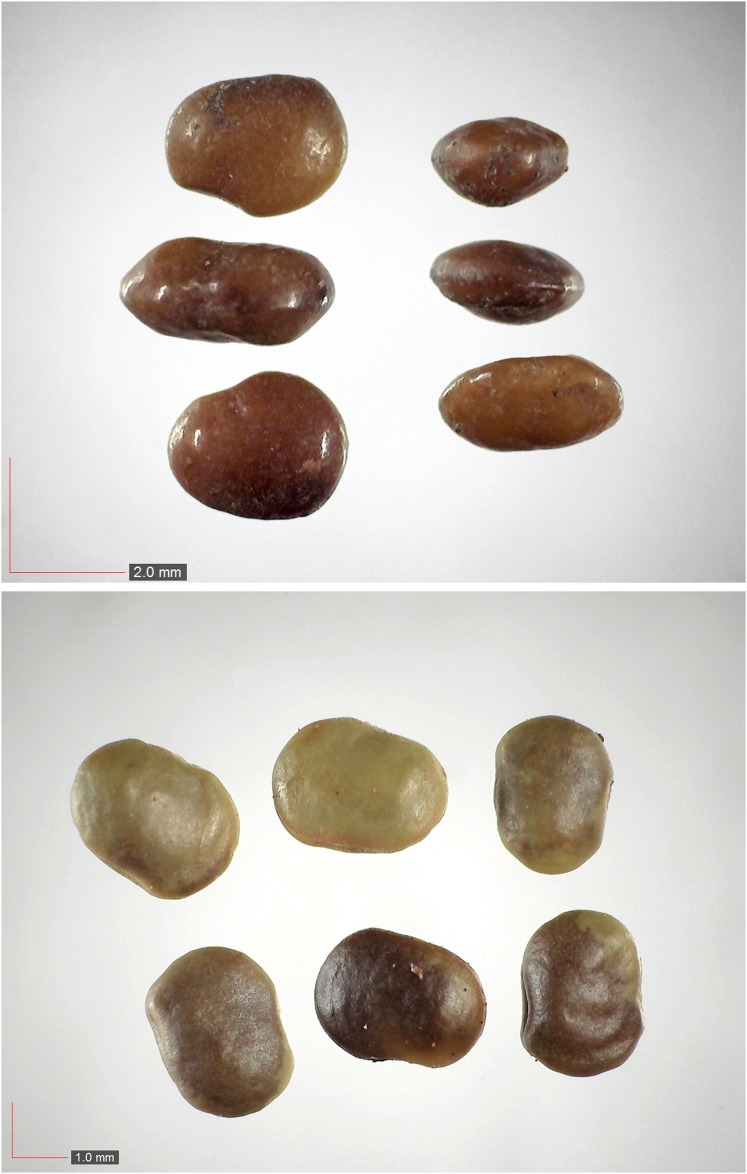
*Rhus copallinum* 0.2% (Anacardiaceae). Top: seeds from *P*. *badius* nest; bottom: seeds from herbarium. Table 2 / AOFP / FSU Herbarium.

**Fig 35:**
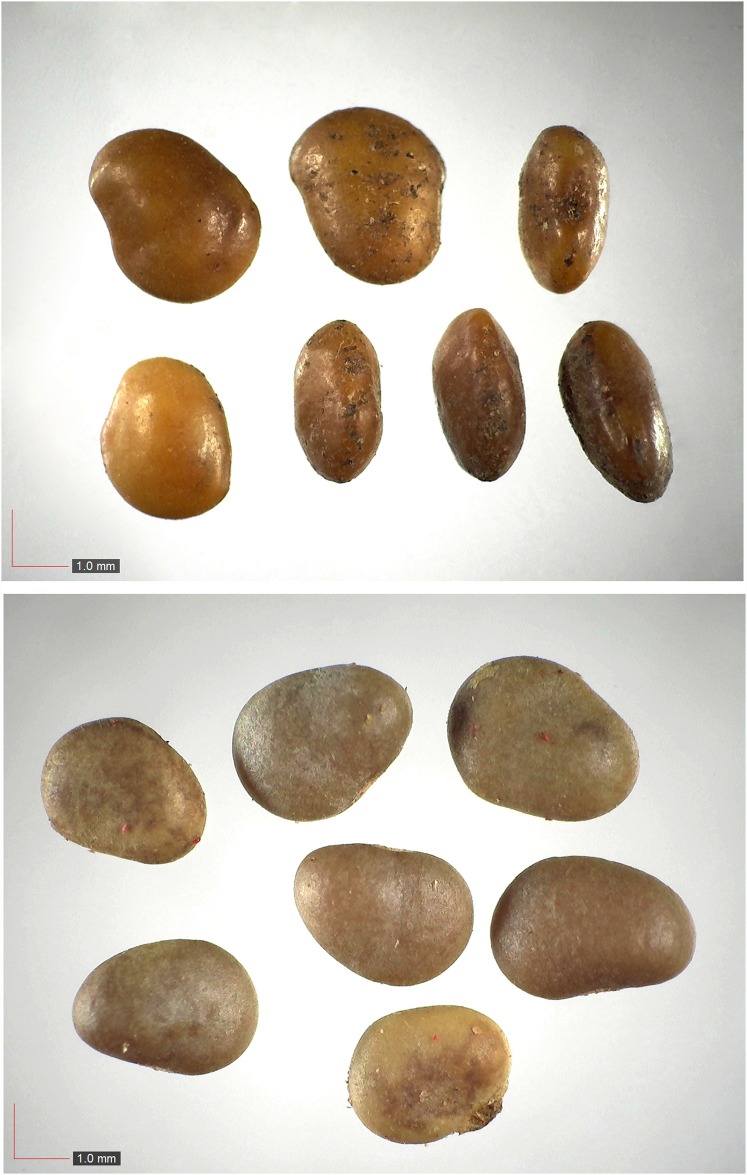
*Rhus glabra* 3.7% (Anacardiaceae). Top: seeds from *P*. *badius* nest; bottom: seeds from herbarium. Table 2 / AOFP / FSU Herbarium.

**Fig 36:**
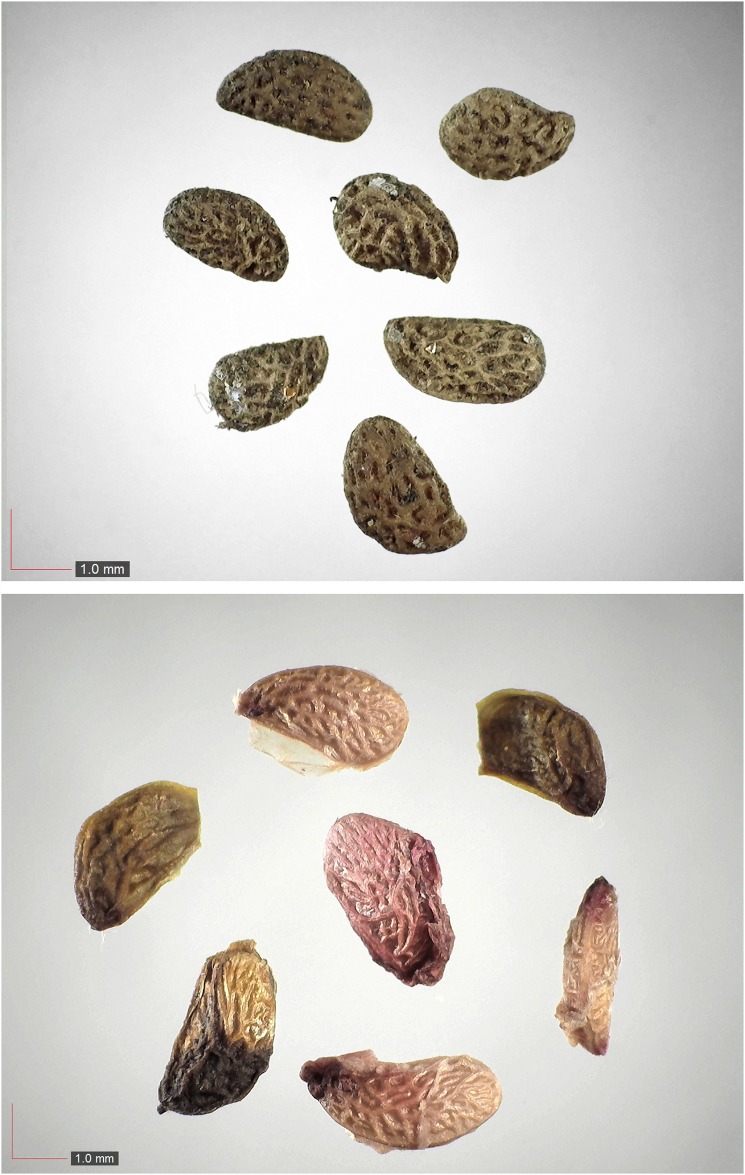
*Rubus trivialis* 0.5% (Rosaceae). Top: seeds from *P*. *badius* nest; bottom: seeds from herbarium. Table 2 / AOFP / FSU Herbarium.

**Fig 37:**
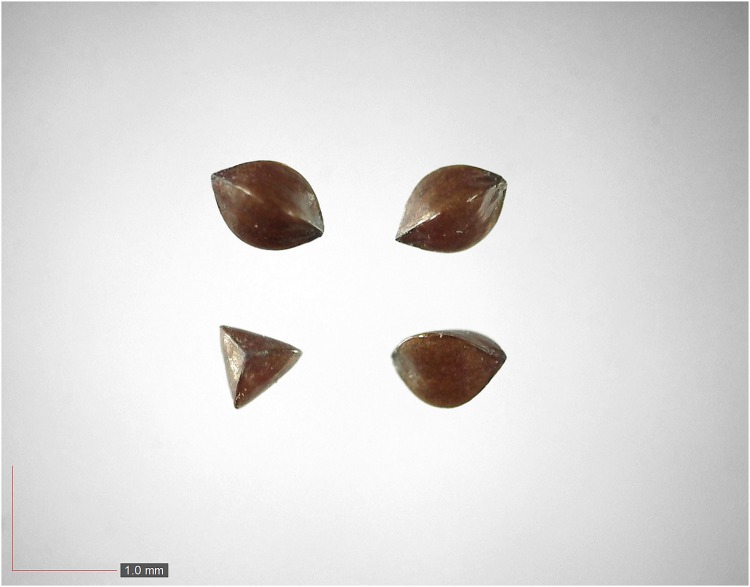
*Rumex hastatulus* (Polygonaceae). Table 2 / AOFP / FSU Herbarium.

**Fig 38:**
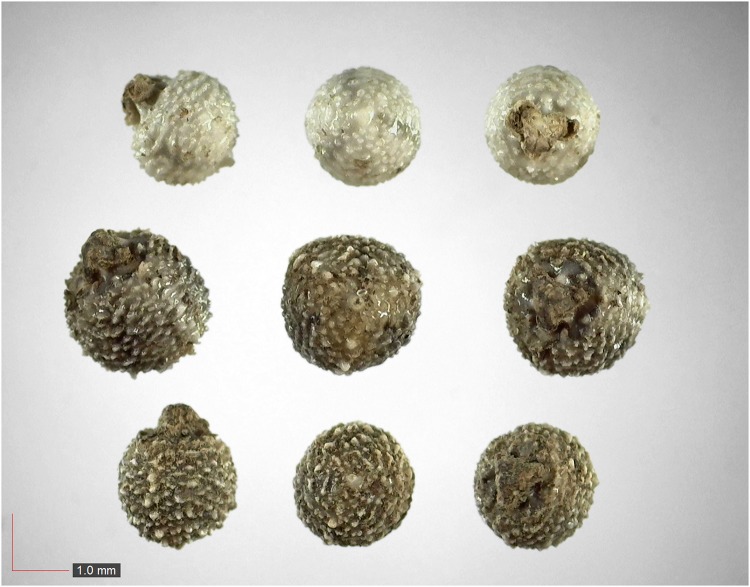
*Scleria sp*. *A* (Cyperaceae). Table 2 / AOFP / FSU Herbarium.

**Fig 39:**
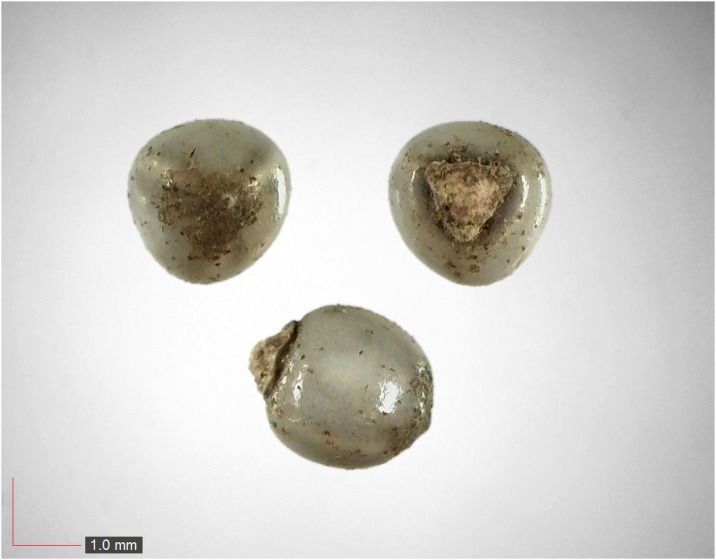
*Scleria sp*. *B* (Cyperaceae). Table 2 / AOFP / FSU Herbarium.

**Fig 40:**
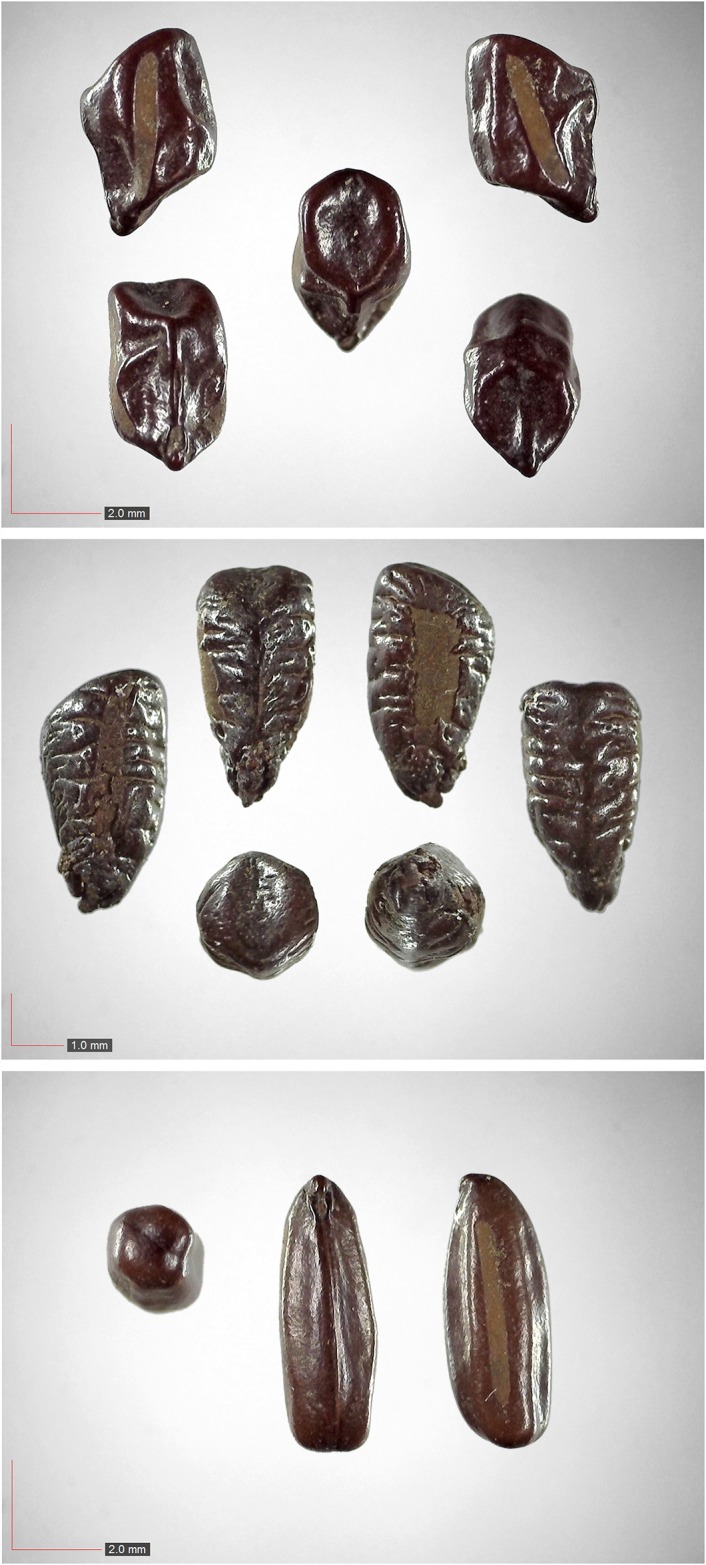
*Senna obtusifolia* 0.0% (Fabaceae). Seed morphology is highly variable, as seen in these three examples. Table 2 / AOFP / FSU Herbarium.

**Fig 41:**
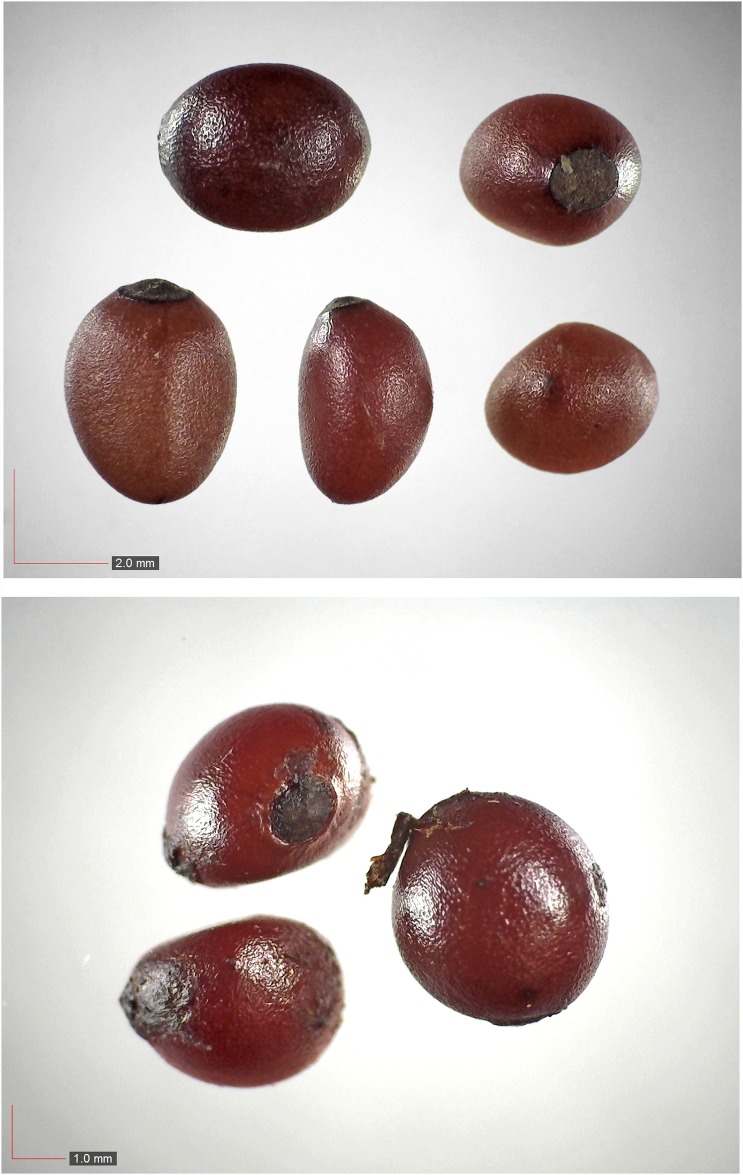
*Smilax auriculata* 0.1% (Smilacaceae). Top: seeds from *P*. *badius* nest; bottom: seeds from herbarium. Table 2 / AOFP / FSU Herbarium.

**Fig 42:**
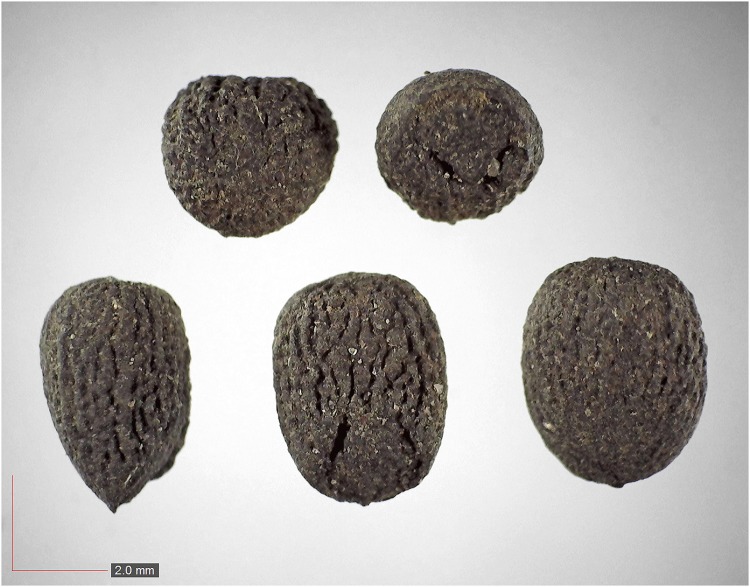
*Stillingia sylvatica* (Euphorbiaceae). Table 2 / AOFP / FSU Herbarium.

**Fig 43:**
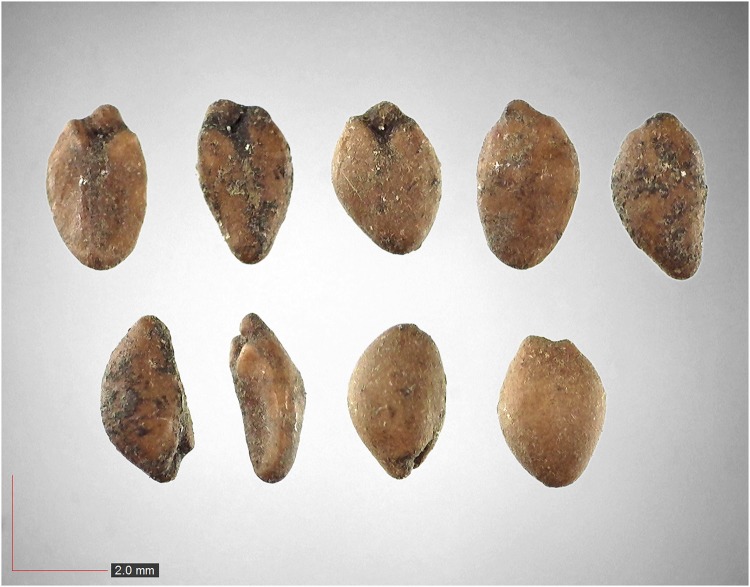
*Stylisma humistrata* 0.2% (Convulvulaceae). Table 2 / AOFP / FSU Herbarium.

**Fig 44:**
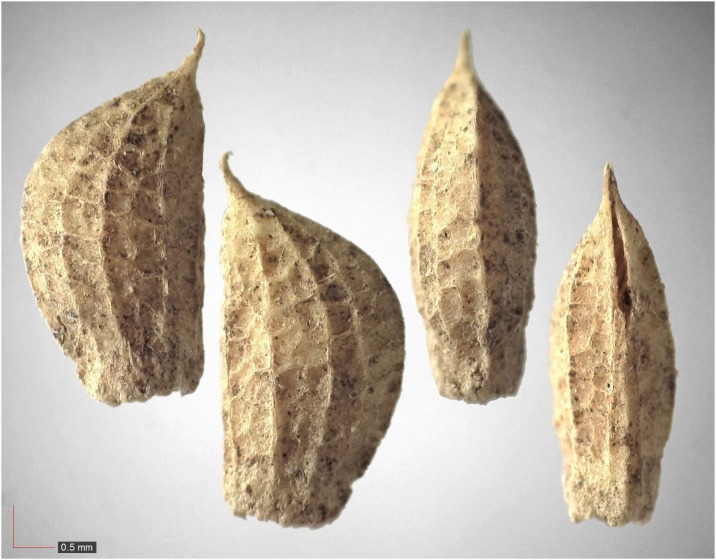
*Stylosanthes biflora* (Fabaceae). Table 2 / AOFP / FSU Herbarium.

**Fig 45:**
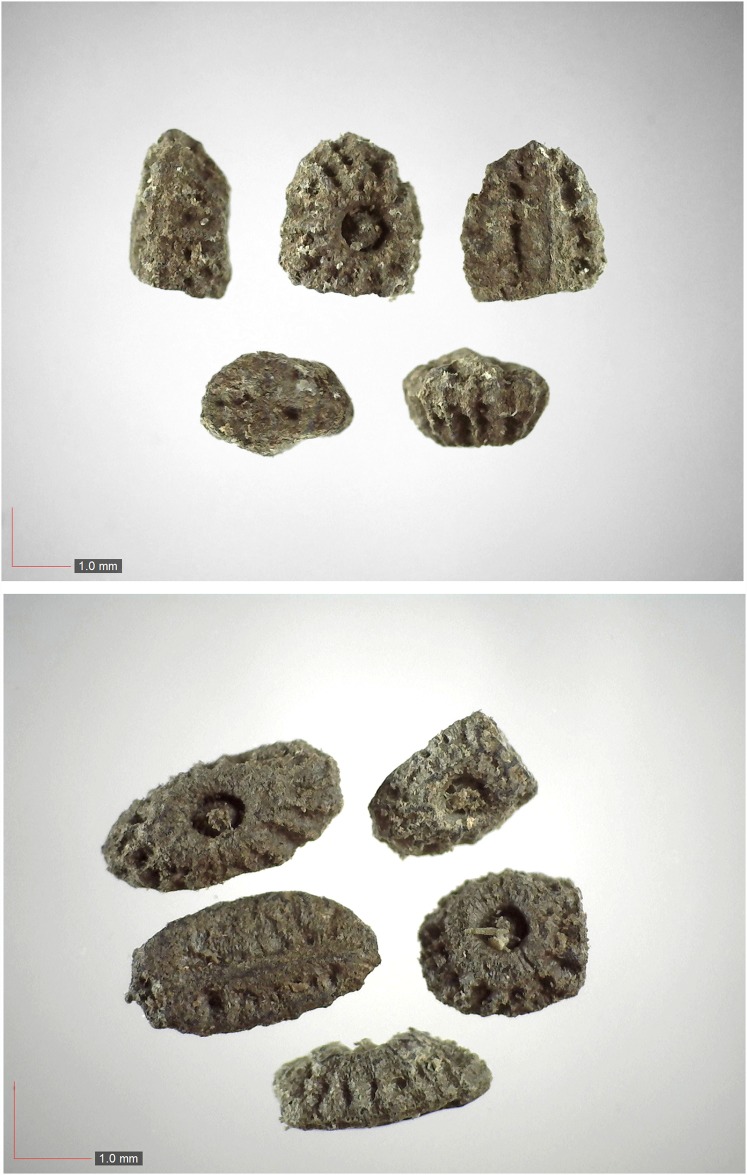
*Tradescantia ohiensis* 0.2% (Commelinaceae). Top: seeds from *P*. *badius* nest; bottom: seeds from herbarium. Table 2 / AOFP / FSU Herbarium.

**Fig 46:**
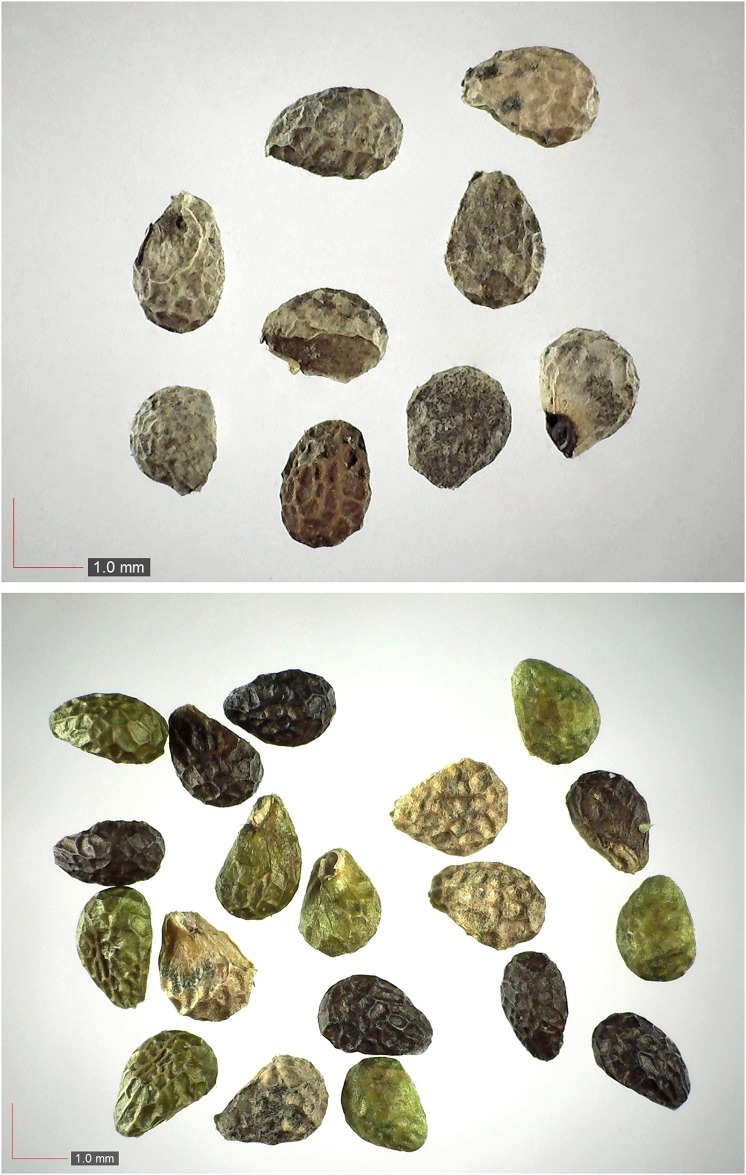
*Trichostema dichotomum* 2.6% (Lamiaceae). Top: seeds from *P*. *badius* nest; bottom: seeds from herbarium. Table 2 / AOFP / FSU Herbarium.

**Fig 47:**
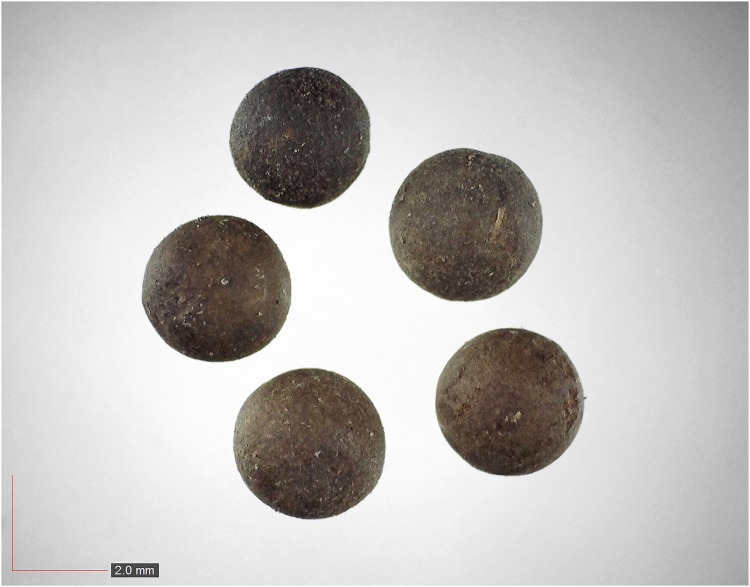
*Vicia sativa –uncertain ID* .5% (Fabaceae). Table 2 / AOFP / FSU Herbarium.

**Fig 48:**
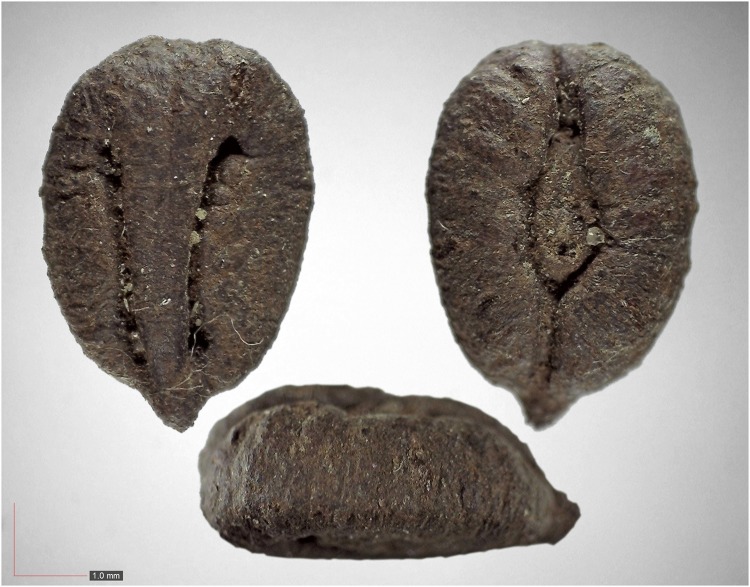
*Vitis rotundifolia* (Vitaceae). Table 2 / AOFP / FSU Herbarium.

**Unidentified seeds**

**Fig 49:**
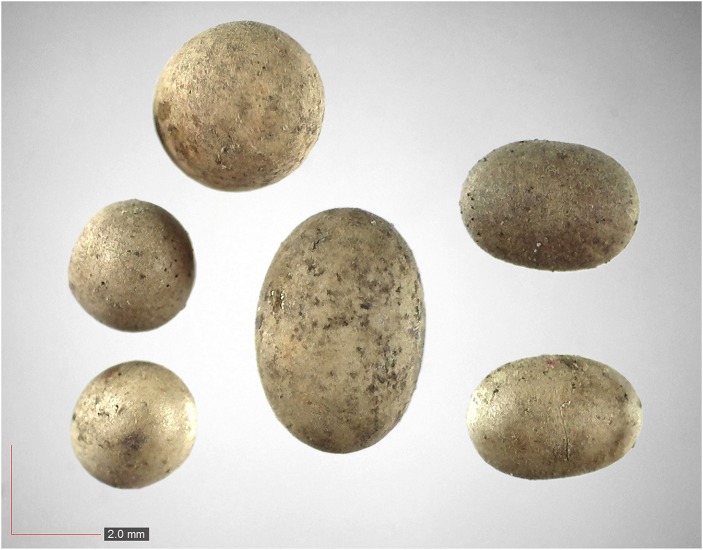
Unidentified 1. Table 2.

**Fig 50:**
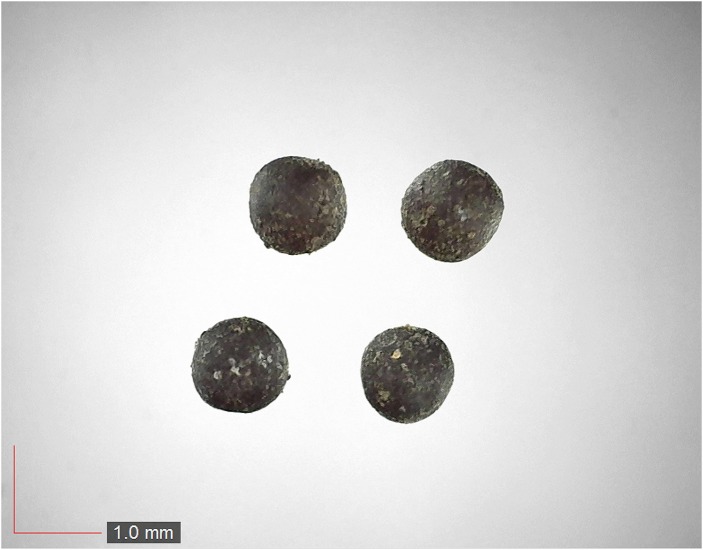
Unidentified 2. Table 2.

**Fig 51:**
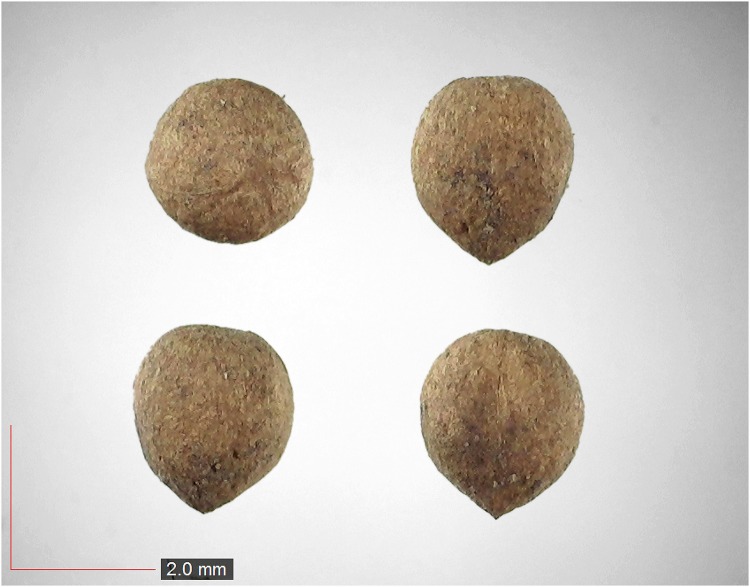
Unidentified 3. Table 2.

**Fig 52:**
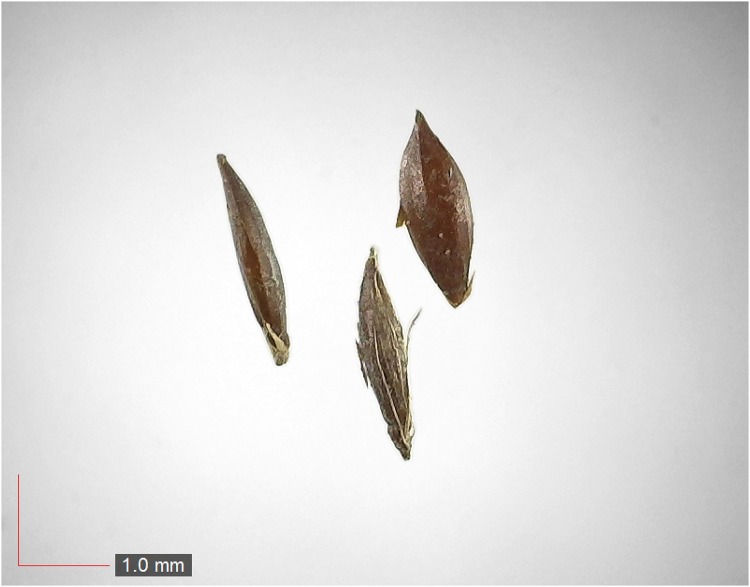
Unidentified 4 (probably *Digitaria*). Table 2.

**Fig 53:**
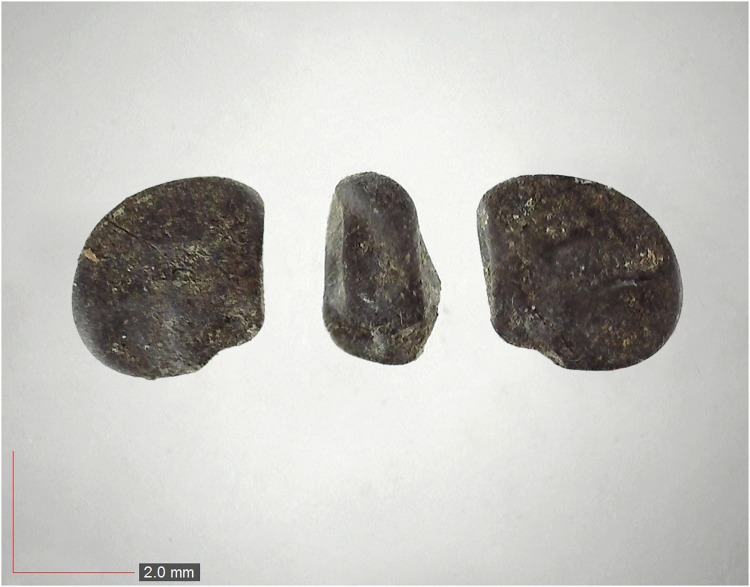
Unidentified 5. Table 2.

**Fig 54:**
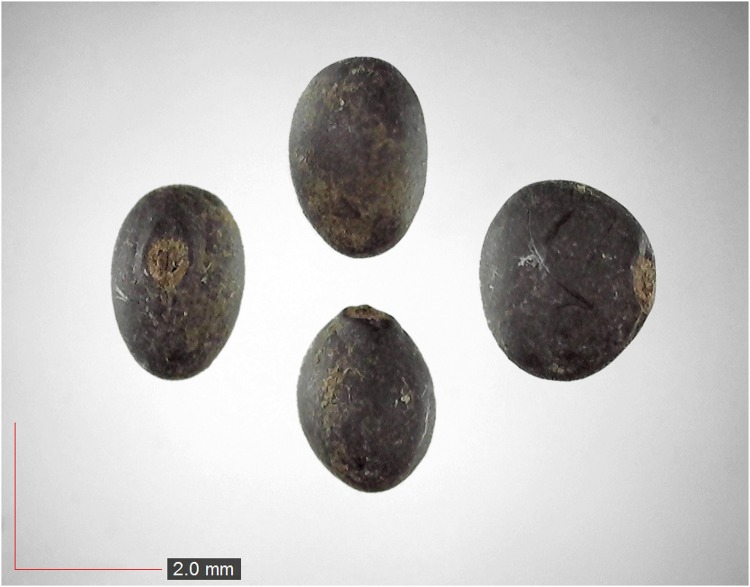
Unidentified 6. Table 2.

**Fig 55:**
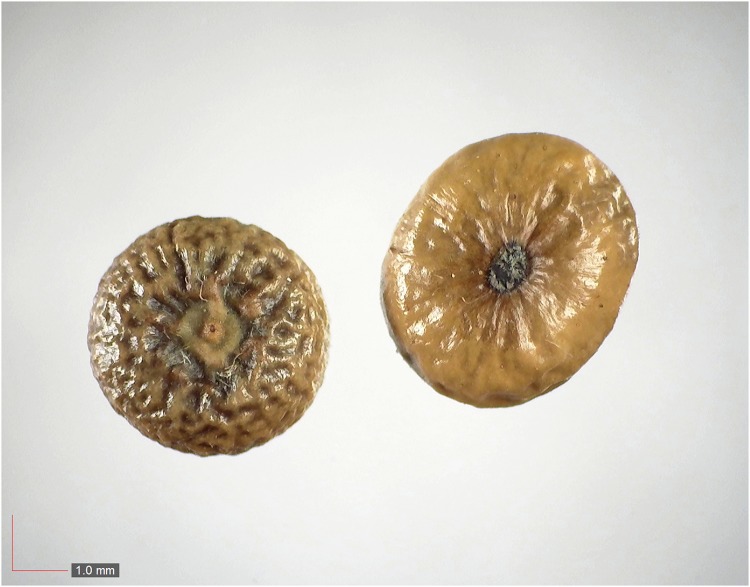
Unidentified 7. Table 2.

**Fig 56:**
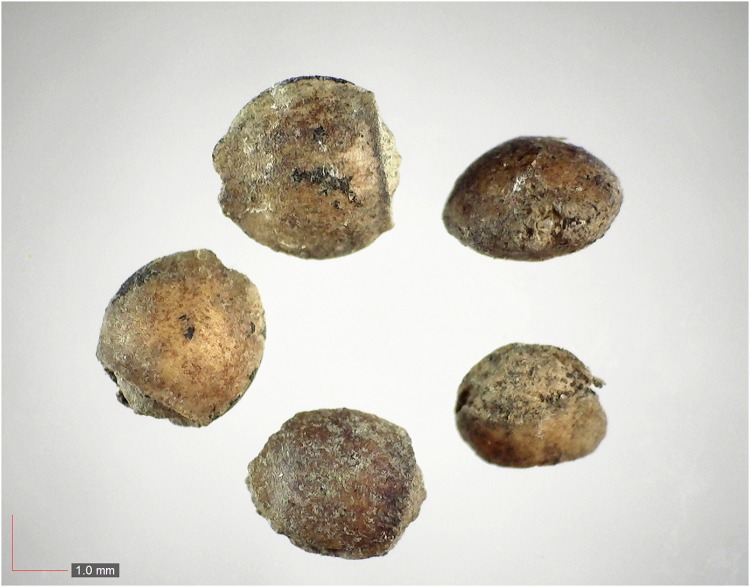
Unidentified 8. Table 2.

**Fig 57:**
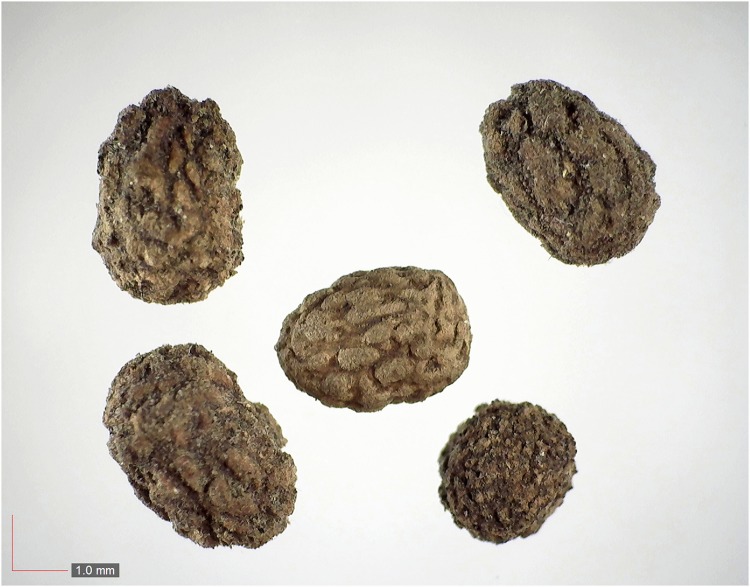
Unidentified 9. Table 2.

**Fig 58:**
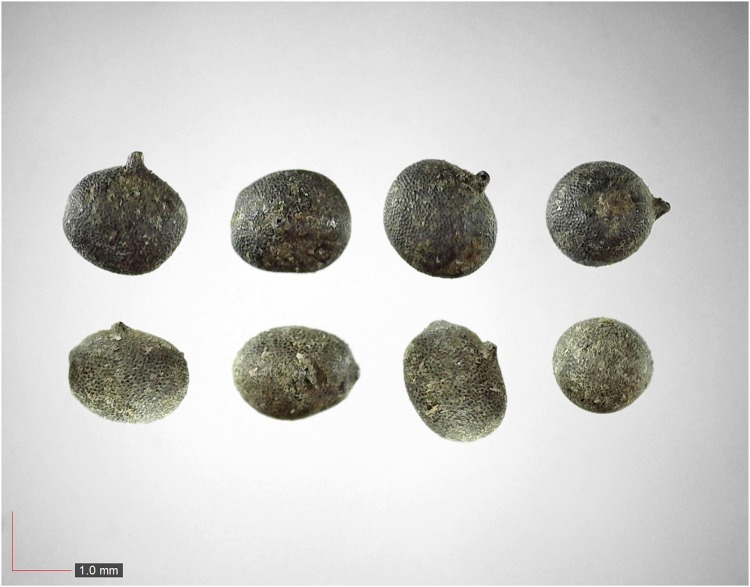
Unidentified 10. Table 2.

Figs 1–58. Seeds found in the nests of the Florida harvester ant, *Pogonomyrmex badius*. The images are linked to Table 1, and are shown in multiple aspects with a scale. Relative weight is also presented for many seeds in Table 1.

Table 3 lists species of seeds found at the study site, Ant Heaven, but not found in *P*. *badius* nests. Images for these seeds can be found in alphabetical order in Figs 59–78. Whether this absence resulted from choices made by ant foragers, seed abundance, season of seed ripening or some other chance factor is unknown. We have made no attempts to estimate the relative availability of ant and non-ant seeds in the landscape (a difficult task, without doubt).

**Table 3:** Seeds found at the study site, Ant Heaven, but not in the nests of harvester ants. Each species name is linked to its image below. These species are only a small sample of those found at Ant Heaven.

Seeds present in field but not found/confirmed in nests	Family	Figure No.
***Agalinis tenuifolia***	Orobanchaceae	Fig 59
***Bulbostylis ciliatifolia***	Cyperaceae	Fig 60
***Carphephorus odoratissimus***	Asteraceae	Fig 61
***Crocanthemum carolinianum***	Cistaceae	Fig 62
***Crotalaria purshii***	Fabaceae	Fig 63
***Elephantopus tomentosus***	Asteraceae	Fig 64
***Froelichia floridana***	Amaranthaceae	Fig 65
***Gelsemium sempervirens***	Gelsemiaceae	Fig 66
***Hieracium gronovii***	Asteraceae	Fig 67
***Hypericum tenuifolium***	Clustaceae	Fig 68
***Ilex glabra***	Ericaceae	Fig 69
***Ilex vomitoria***	Ericaceae	Fig 70
***Liatris tenuifolia***	Asteraceae	Fig 71
***Lupinus villosus***	Fabaceae	Fig 72
***Palafoxia integrifolia***	Asteraceae	Fig 73
***Penstemon multiflorus***	Scrophalariaceae	Fig 74
***Polypremum procumbens***	Tetrachondraceae	Fig 75
***Sabatia brevifolia***	Gentianaceae	Fig 76
***Sericocarpus tortifolius***	Asteraceae	Fig 77
***Seymeria cassioides***	Orobanchaceae	Fig 78

**Fig 59:**
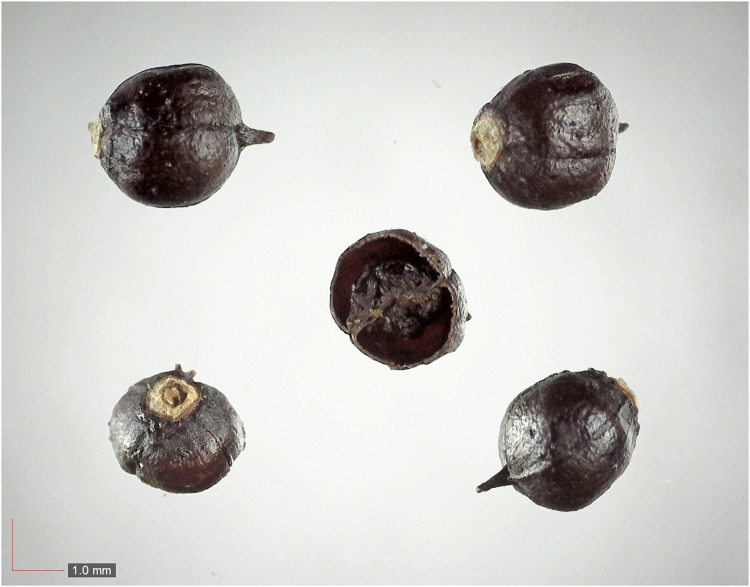
*Agalinis tenuifolia* (Orobanchaceae) Herbarium seed. Table 3 / AOFP / FSU Herbarium.

**Fig 60:**
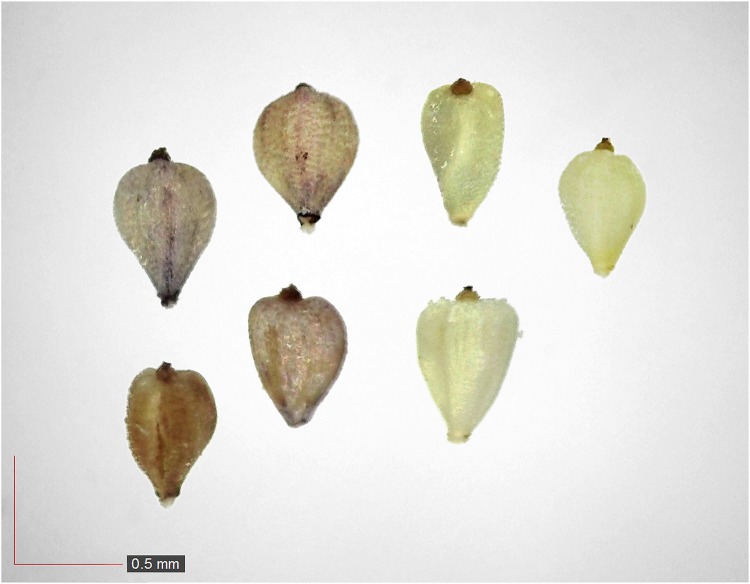
*Bulbostylis ciliatifolia* (Cyperaceae) Field seed. Table 3 / AOFP / FSU Herbarium.

**Fig 61:**
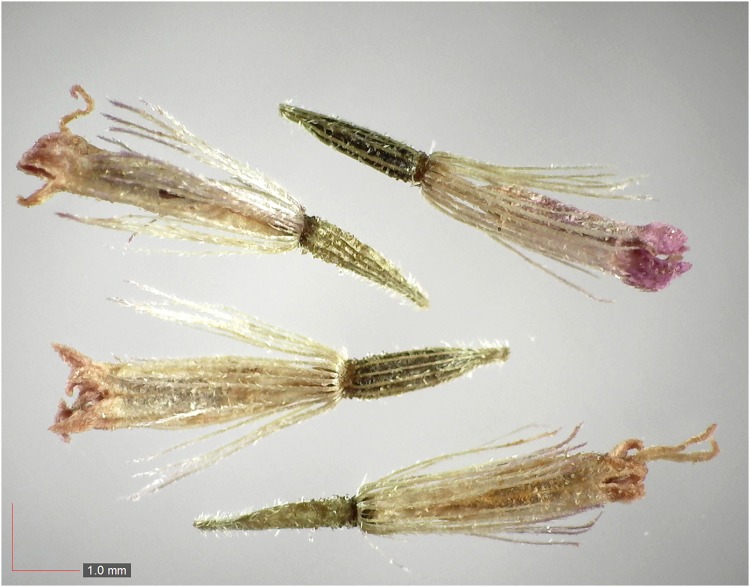
*Carphephorus odoratissimus* (Asteraceae) Herbarium seed. Table 3 / AOFP / FSU Herbarium.

**Fig 62:**
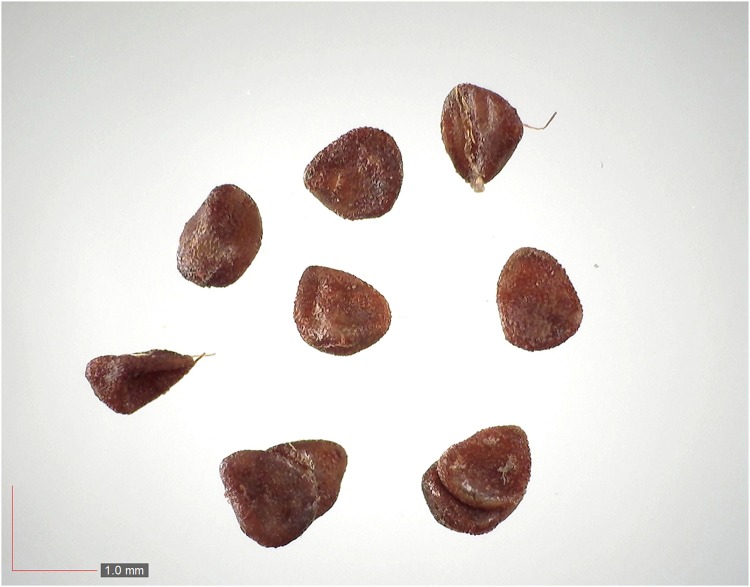
*Crocanthemum carolinianum* (Cistaceae) Field seed. Table 3 / AOFP / FSU Herbarium.

**Fig 63:**
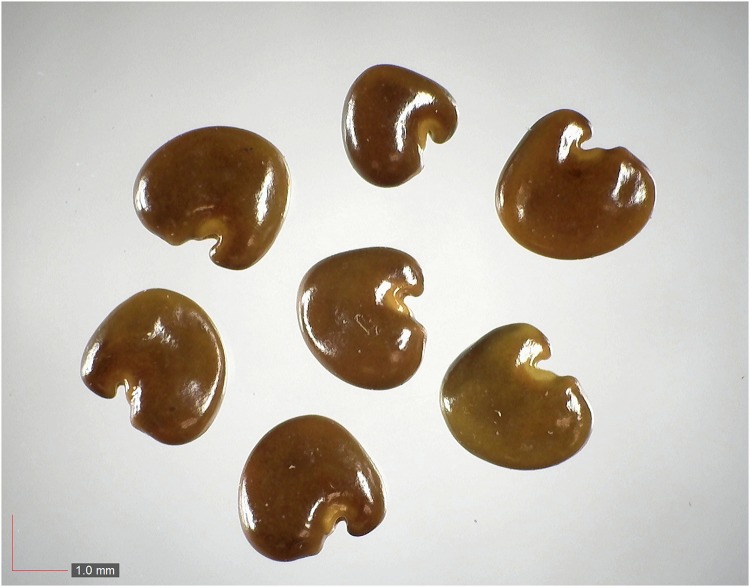
*Crotalaria purshii* (Fabaceae) Herbarium seed. Table 3 / AOFP / FSU Herbarium.

**Fig 64:**
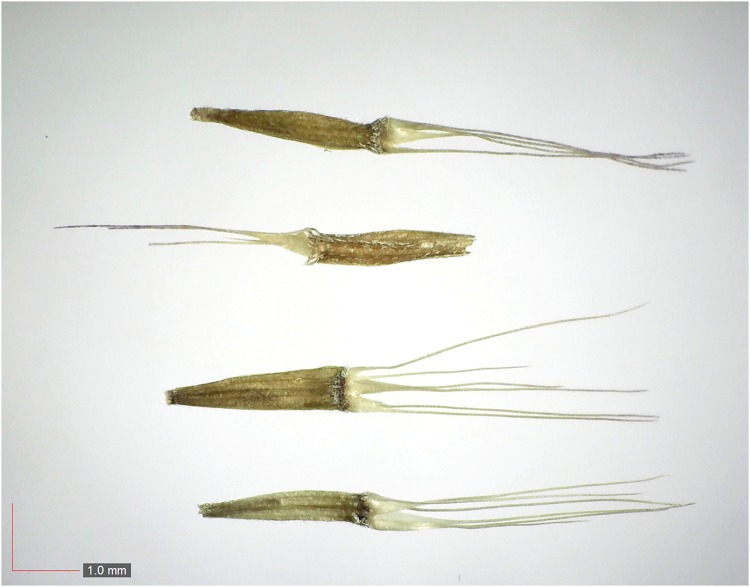
*Elephantopus tomentosus* (Asteraceae) Herbarium seed. Table 3 / AOFP / FSU Herbarium.

**Fig 65:**
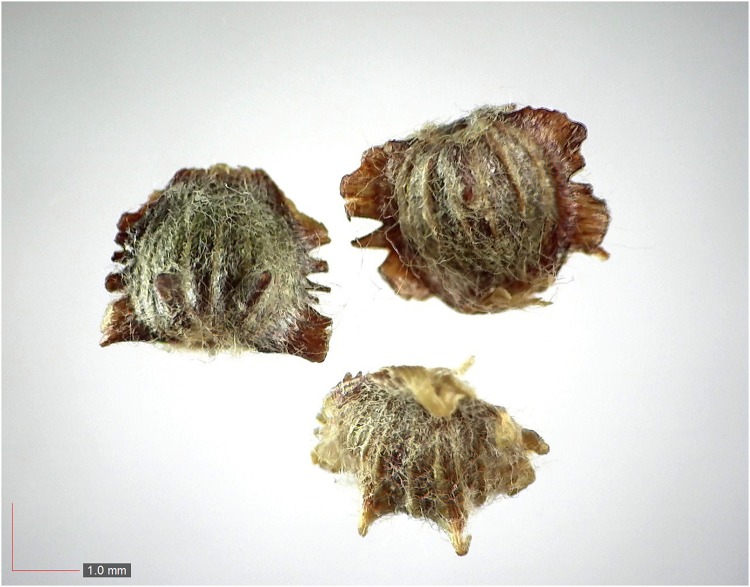
*Froelichia floridana* (Amaranthaceae) Herbarium seed. Table 3 / AOFP / FSU Herbarium.

**Fig 66:**
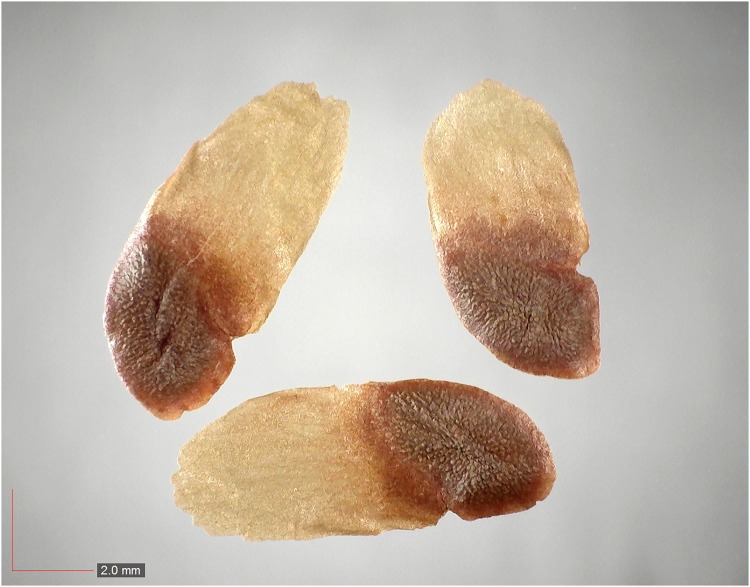
*Gelsemium sempervirens* (Gelsemiaceae) Field seed. Table 3 / AOFP / FSU Herbarium.

**Fig 67:**
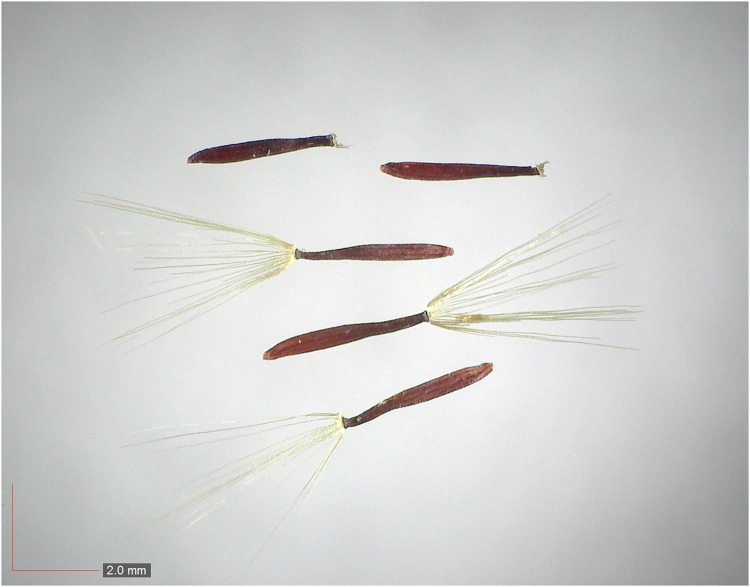
*Hieracium gronovii* (Asteraceae) Herbarium seed. Table 3 / AOFP / FSU Herbarium.

**Fig 68:**
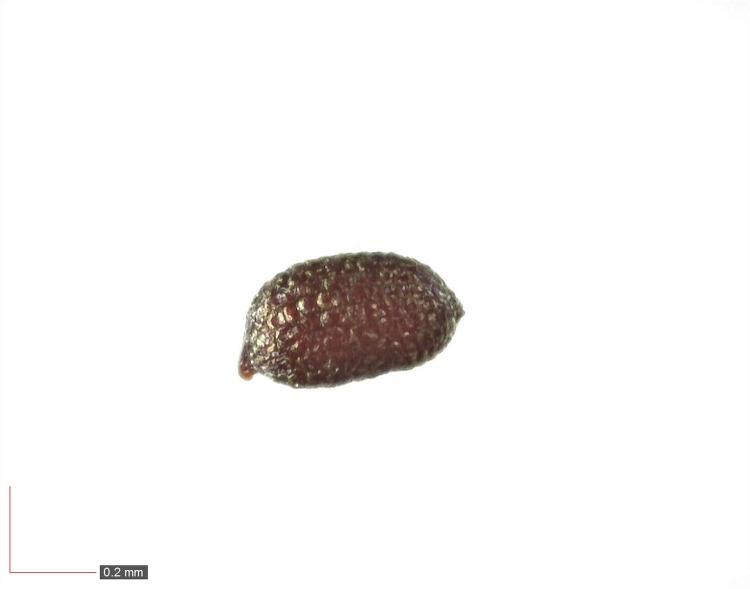
*Hypericum tenuifolium* (Clustaceae) Herbarium seed. Table 3 / AOFP / FSU Herbarium.

**Fig 69:**
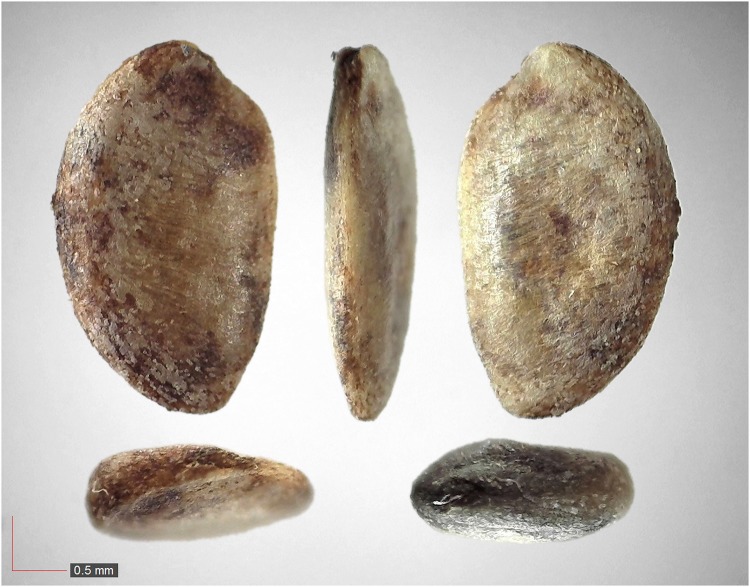
*Ilex glabra* (Ericaceae) Field seed. Table 3 / AOFP / FSU Herbarium.

**Fig 70:**
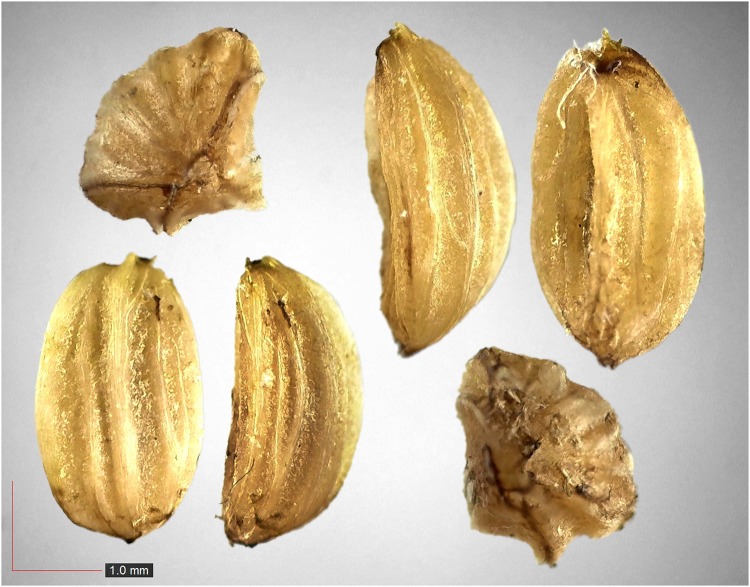
*Ilex vomitoria* (Ericaceae) Field seed. Table 3 / AOFP / FSU Herbarium.

**Fig 71:**
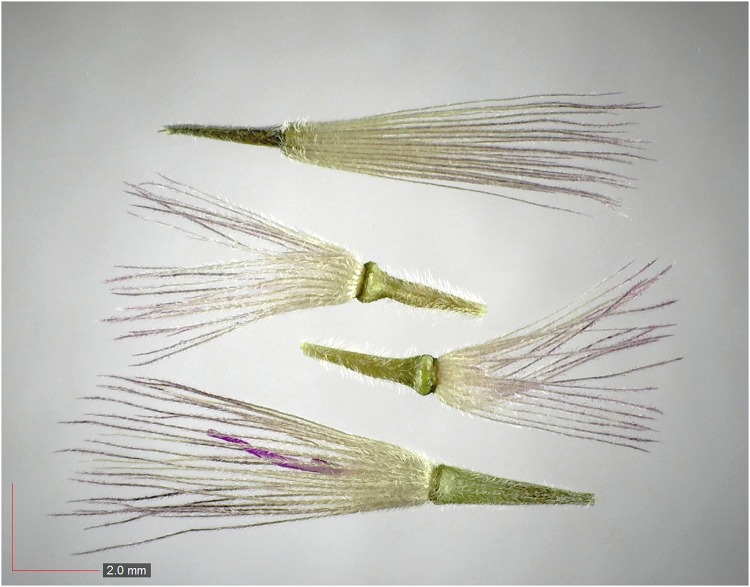
*Liatris tenuifolia* (Asteraceae) Herbarium seed. Table 3 / AOFP / FSU Herbarium.

**Fig 72:**
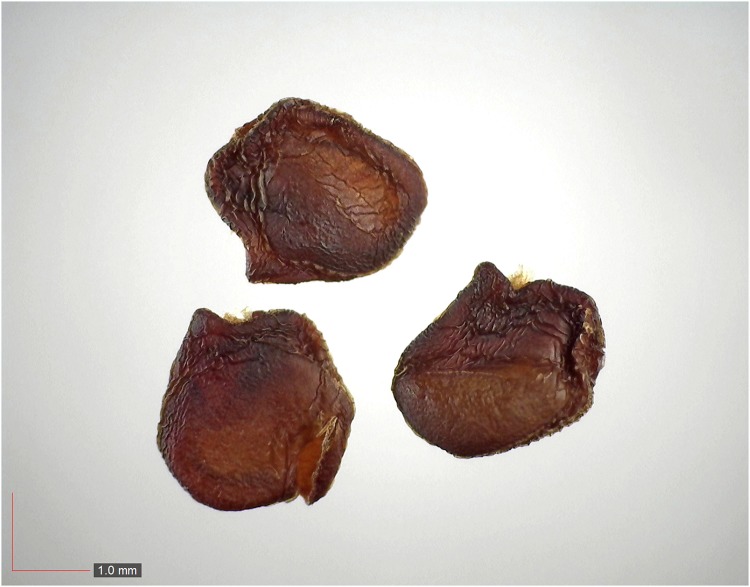
*Lupinus villosus* (Fabaceae) Herbarium seed. Table 3 / AOFP / FSU Herbarium.

**Fig 73:**
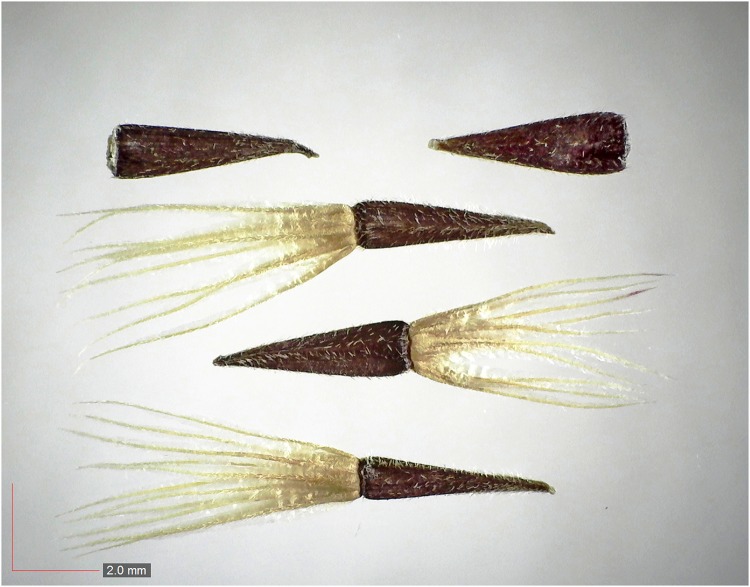
*Palafoxia integrifolia* (Asteraceae) Herbarium seed. Table 3 / AOFP / FSU Herbarium.

**Fig 74:**
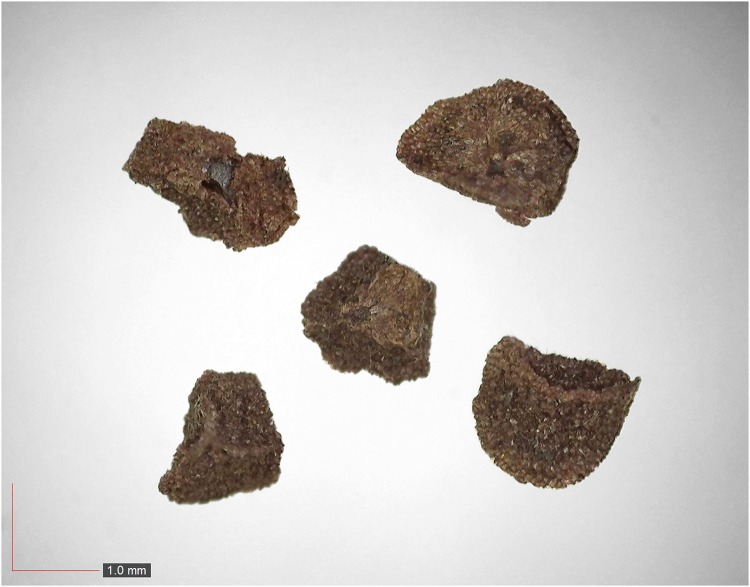
*Penstemon multiflorus* (Plantaginaceae) Herbarium seed. Table 3 / AOFP / FSU Herbarium.

**Fig 75:**
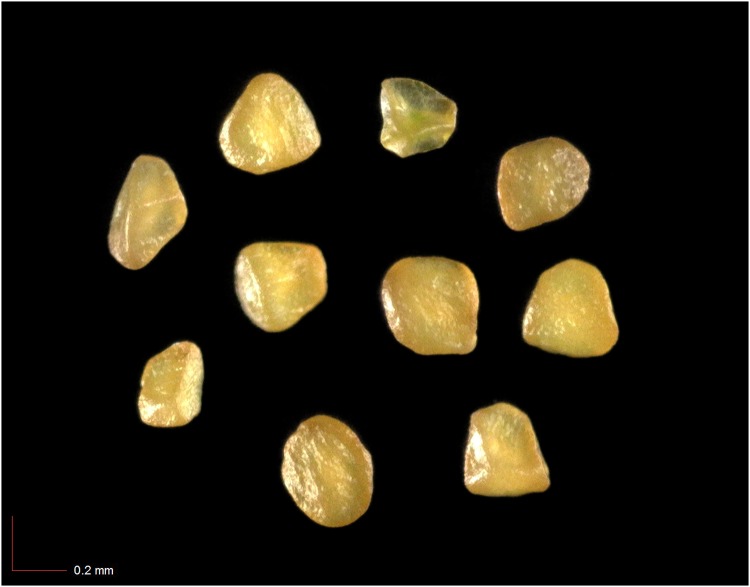
*Polypremum procumbens* (Tetrachondraceae) Herbarium seed. Table 3 / AOFP / FSU Herbarium.

**Fig 76:**
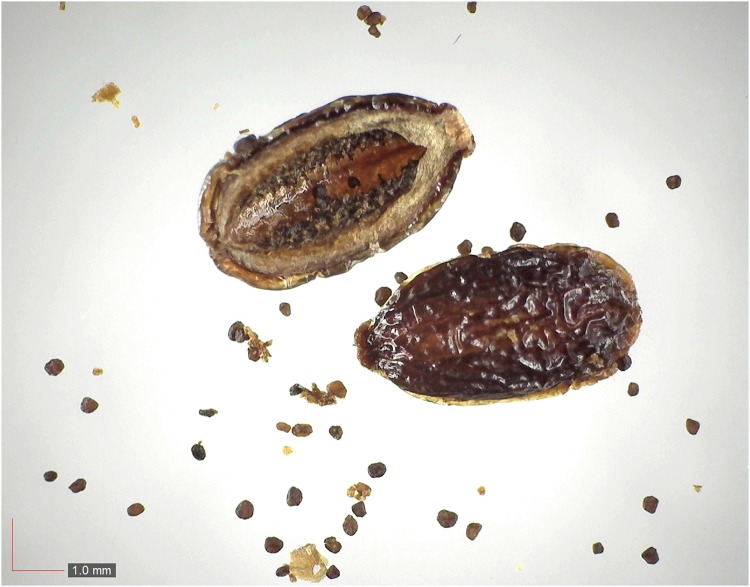
*Sabatia brevifolia* (Gentianaceae) Herbarium seed. Table 3 / AOFP / FSU Herbarium.

**Fig 77:**
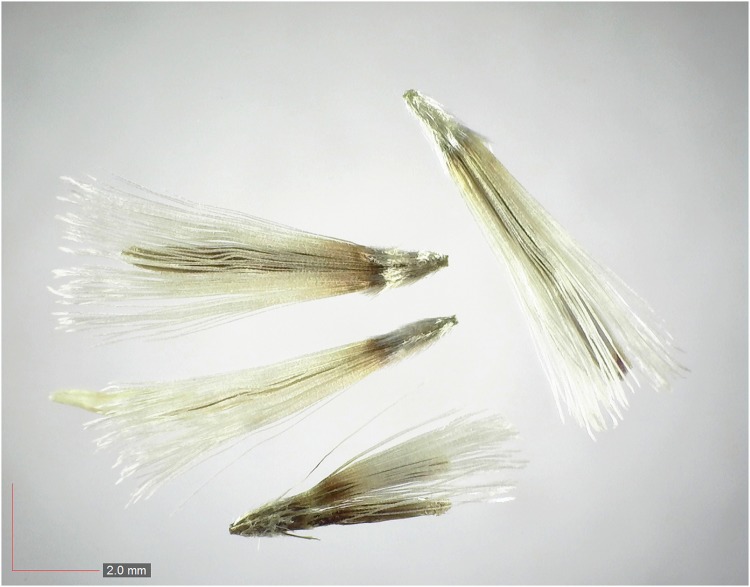
*Sericocarpus tortifolius* (Asteraceae) Herbarium seed. Table 3 / AOFP / FSU Herbarium.

**Fig 78:**
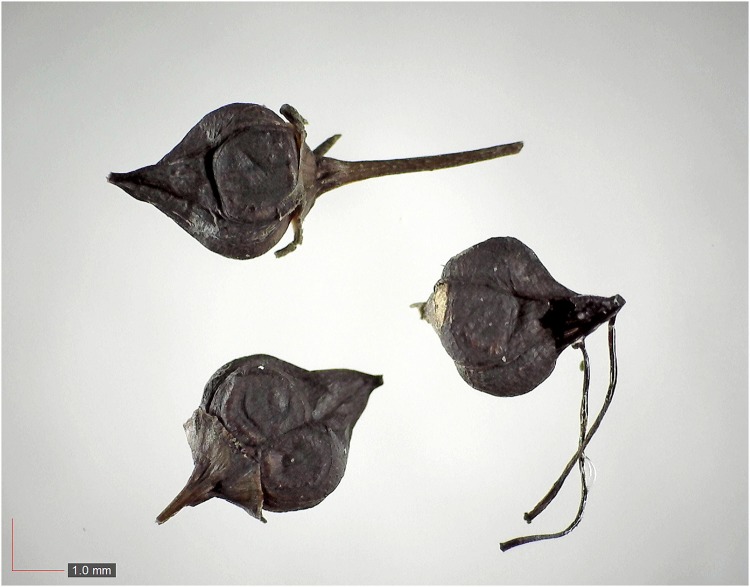
*Seymeria cassioides* (Orobanchaceae) Herbarium seed. Table 3 / AOFP / FSU Herbarium.

Figs 59–78. Seeds not found in harvester ant nests. Field seeds were collected at the study site, Ant Heaven. Herbarium seeds were taken from dried herbarium specimens of plants occurring at Ant Heaven. (R.K. Godfrey Herbarium at Florida State University).

The number of species per plant family ranged from ten (Fabaceae) to one (10 families) (Table 4). Ten families were represented by two to five species, and another ten by a single species. Some of the singleton families are either rare at the site, or represented by only one or two species (e.g. Cactaceae, Smilacaceae), but others are common and/or speciose at the site, yet infrequent or absent in the nest stores. For example, the Asteraceae are represented by a substantial number of species, some fairly common, yet seeds of Asteraceae are uncommon in the seed stores. On the other hand, *Ilex glabra* (gallberry) and *Gaylussacia dumosa* (shiny blueberry) are common shrubs at the site, but their seeds are infrequent in *P*. *badius* nests.

**Table 4:** Species distribution among families. Seed species represented 20 plant families, with the most species from the Fabaceae, followed by Poaceae. Ten families were represented by more than one species, and 12 by a single species each.

Family	Number of species in *P*. *badius* nests	Number of species NOT in nests, but at site
Fabaceae	10	2
Poaceae	5	0
Ephorbiaceae	3	0
Polygonaceae	3	0
Cyperaceae	3	1
Pinaceae	3	0
Anacardiaceae	2	0
Commelinaceae	2	0
Convulvulaceae	2	0
Ericaceae	2	2
Lamiaceae	1	0
Rubiaceae	1	0
Rosaceae	1	0
Vitaceae	1	0
Cactaceae	1	0
Smilacaceae	1	0
Asteraceae	1	6
Clustaceae	1	1
Cornaceae	1	0
Phytolaccaceae	1	0
Orobanchaceae	0	2
Cistaceae	0	1
Amaranthaceae	0	1
Gelsemiaceae	0	1
Scrophalariaceae	0	1
Tetrachondraceae	0	1
Gentianaceae	0	1

## Discussion

The seeds of more than a dozen plant species occur frequently in the nests of the Florida harvester ant, with another almost four dozen occurring occasionally to rarely. The 58 species we found in nests represent an important food resource for the ants, but the presence at Ant Heaven of seed species that were not found in *P*. *badius* nests suggests some degree of either selectivity by foragers or availability in the habitat. Whereas cafeteria experiments with Ant Heaven seeds might reveal forager preferences among seed species, it is difficult to test if the relative abundance in nest chambers reflects availability, for to do so, one must see seeds through the eyes of foragers, not humans. What may seem available to a human may or may not be available to an ant and vice versa.

The 58 species found in nests belonged to 20 plant families, several represented by multiple species, but an additional seven families were found at the site but not in nests. Because our cataloguing was opportunistic, these seven families represent only a fraction of the plant families occurring at the site. In contrast, the 20 families represented in the nest stores are probably a large fraction of the total that an enlarged search would reveal. This uneven representation of seeds from a range of plant families poses questions about the suitability of seeds as ant food, and subsequently, whether the ants have evolved a preference for some species and an avoidance of others. Considering the importance of germination for gaining access to seeds as food, the phenology of production and germination may be important. It is particularly striking that ten species of Fabaceae and five Poeaceae were found in ant nests, with only two and zero (respectively) found at the site but not in nests. On the other hand, only a single species of Asteraceae occurred in nests, but six were present at the site but not in nests. Also striking is that although both gallberry (*Ilex glabra*) and shiny blueberry (*Gaylussacia dumosa*) were extremely abundant at the site, they were infrequent to rare in the nest stores. The tiny seeds of the very abundant dog fennel (*Eupatorium capillifolium*) were completely absent from nest stores, as were those of beardgrass (*Andropogon* sp.).

A few species of seeds were obviously not within the foraging range of the *P*. *badius* nests at Ant Heaven. Neither of the species of *Magnolia* grew there, nor did *Nyssa sylvatica*, *Phytolacca americana* or *Ilex myrtifolia*. A possible source of importation might be animal feces, but this remains to be evaluated.

This study was carried out on one population at one site. Vegetation, and therefore seed inventory, almost surely differs among sites. To the degree to which seed stores show or do not show consistency across sites, site differences could illuminate the question of availability vs. preference. No doubt, other researchers will add to the list of seeds stored in *P*. *badius* nests, along with geographic and ecological information. The high quality, multiple-aspect images we present here will make future identification of seeds from harvester ant nests much easier, and may stimulate more studies.

**Fig 79:**
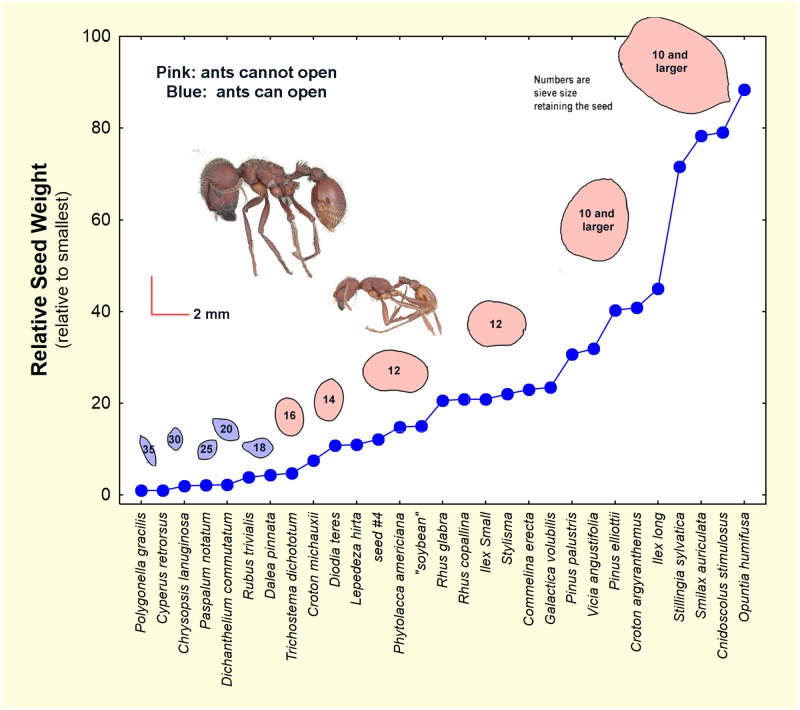
The relative weights of seeds (relative to the smallest) and sizes of ants. Silhouettes show seeds from *P*. *badius* nests that the ants are able to open (blue) and unable to open (pink). *P*. *badius* major and minor workers are shown to the same scale. Ants can open only the smallest of this array of seeds. Details can be found in Tschinkel and Kwapich [21]. The seed list is not complete, but is representative of the total. Seed silhouette positions on the abscissa are in approximate order of their relative weights. The number in each seed silhouette is the U.S. Standard testing sieve on which the seed was retained. Ant images modified from antweb.org. Photos by April Nobile: minor worker:URL: https://www.antweb.org/bigPicture.do?name=casent0104423&shot=p&number=1. Major worker: https://www.antweb.org/bigPicture.do?name=casent0103057&shot=p&number=1.

Because the seeds are ant food, their size and nutritional quality are important attributes. We lack information on quality, but Fig 79 dramatizes the nutritional, caloric benefit of exploiting larger seeds. Relative to the smallest seeds found in *P*. *badius* nests, larger seeds provide up to 90-fold more food. However, the ants cannot open seeds larger than about 1 to 1.4 mm across [21]. Tschinkel and Kwapich [21] showed that large seeds are used once they germinate in the nest, and the ants benefit greatly from this strategy. For example, a germinating *Smilax auriculata* seed (which germinate fairly regularly) would yield 70 times as much food as an ant-opened *Polygonella gracilis* seed. In view of the benefits of this strategy, it seems inevitable that it would evolve.

*P*. *badius* is only one of many ant species that harvest seeds and store them for later consumption. Several species of ants collect multiple species of seeds, but it remains to be determined whether other species also exploit seed germination to increase the range of seed sizes and species they are able to consume.
